# Histopathological examination and transcriptomic profiling reveal gossypol toxicity-responsive genes related to fertility in mice

**DOI:** 10.3389/fphar.2025.1654299

**Published:** 2025-08-29

**Authors:** Teame Gereziher Mehari, Maorong Jiang, Dandan Gu, Jiaxi Qian, Jungfeng Tang, Ashenafi Kiros Wubshet, Hui Fang, Kai Wang, Deng-Bing Yao, Baohua Wang

**Affiliations:** ^1^ School of Life Sciences, Nantong University, Nantong, China; ^2^ Key Laboratory of Neuroregeneration of Jiangsu and Ministry of Education, School of Life Sciences, Co-Innovation Center of Neuroregeneration, Nantong University, Nantong, China; ^3^ Department of Cell Engineering and Biotechnology, Shandong Province Binzhou Animal Husbandry and Veterinary Institute, Binzhou, China

**Keywords:** gossypol, mice, dimethyl sulfoxide, fertility, transcriptome, *Dmrt2*

## Abstract

**Introduction:**

Gossypol impairs reproduction in both sexes, inducing mitochondrial dysfunction, irregular cycles, and fewer ovarian follicles, leading to decreased fertility rates. The present investigation was undertaken to evaluate the toxicological effects of (+)-gossypol powder, a substance derived from cottonseed in mice.

**Methods:**

The mice were divided into four groups: a control group receiving pure dimethyl sulfoxide (DMSO) and three treatment groups receiving 20, 40, or 80 mg/kg gossypol dissolved in DMSO. The mice were treated daily via oral gavage for 14 days and subjected to clinical observation. At study completion, physical, hematological, and histopathological (ovary, testis, liver, lung, kidney, and spleen) evaluations were performed.

**Results:**

Compared with the control treatment, gossypol treatment adversely affected routine blood parameters and histopathology (p < 0.05). Routine blood tests revealed significantly elevated neutrophil granulocytes and monocytes, along with a marked decrease in lymphocytes. Furthermore, gossypol treatment inhibited spermatogenesis, caused follicular degeneration in the ovaries, and induced tissue damage in the liver, kidney, lung, and spleen. Furthermore, transcriptome sequencing of ovaries, testes, and livers from mice treated with DMSO or 80 mg/kg gossypol was performed to investigate the molecular mechanisms of reproductive damage. Eighteen cDNA libraries were constructed and yielded 113.6 Gb of raw reads, which were filtered to obtain 112.4 Gb of high-quality clean reads. The analysis identified 639 differentially expressed genes (DEGs). Consecutively, weighted gene coexpression network analysis (WGCNA) revealed a strong correlation between the blue module hub gene *ENSMUSG00000048138* (*Dmrt2*) and mouse blood test results. *Dmrt2* gene expression was confirmed by RT-qPCR and GFP expression analysis. Lentivirus-mediated *DMRT2* silencing (shRNA-Sh-a, Sh-b, and Sh-c) resulted in low *DMRT2* expression, whereas overexpression (OE-*DMRT2*) resulted in high *DMRT2* expression relative to that of the controls. Gossypol stress significantly upregulated *Dmrt2* expression in ovarian tissue.

**Conclusion:**

Gossypol powder from cotton is toxic to mice, affecting their pathology, histology, and hematology. This study strongly links the *Dmrt2* gene in reproductive tissues to gossypol resistance, highlighting its potential protective role and advancing the understanding of gene regulation in mouse fertility. Further research and food safety considerations are therefore crucial.

## 1 Introduction

Cotton has been cultivated for more than 7,000 years, primarily to produce cotton fiber. Terpenes, fatty acids, phenolics, carbohydrates, lipids, and proteins can all be found in entire cotton plants and have the potential to be sources of these healthy compounds (Egbuta et al., 2017). It is a widely valued and significant economic crop that provides a significant amount of cottonseeds rich in protein and oil as well as the top natural fiber for the textile industry (Kong et al., 2023). As a result, it significantly contributes to the nutritional requirements and general wellbeing of both humans and livestock (Mehari et al., 2023). However, gossypol, which is poisonous to people and animals with monogastric stomachs, makes oil or protein from cottonseeds inedible (Janga et al., 2019). Secondary metabolism is vital in plant adaptation to the environment because it mediates biointeractions and protects plants against insects, herbivores, and pathogens (Li et al., 2023). Terpenoids are a class of secondary metabolites found in plants in a variety of forms. They are essential for plant growth and development, environmental response, and physiological activities (Yang et al., 2020). Terpenoids have also been widely used in the food, pharmaceutical, and cosmetic industries. They have anticancer, antibacterial, anti-inflammatory, antiviral, and antimalarial properties, as well as the ability to promote transdermal absorption, prevent and treat cardiovascular disease, and have hypoglycemic properties (Yang et al., 2020).

Cotton plants naturally produce certain compounds called sesquiterpenoids, specifically gossypol and hemigossypolone. These compounds have various properties that can fight against insects, fungal, and bacterial infections. They are effective at protecting cotton plants from different types of plant-eating insects, including cotton bollworms and beet armyworms. Gossypol and hemigossypolone are natural compounds found in different parts of cotton plants, with gossypol being the primary phytoalexin in seeds and hemigossypolone being more prevalent in leaves (Huang et al., 2021). It is a yellowish secondary metabolite found in *Gossypium* species and plays a key role in protecting cotton plants and also used as an important anticancer and male contraceptive element (Kong et al., 2023; Tian et al., 2018). It is a significant pigment in cottonseed and poses a significant challenge to the use of cottonseed as a byproduct and to the processing of the seed. It discolours the oil and reacts with protein, lowering the nutritional value of cottonseed products (Agarwal et al., 2003). It is harmful to various biological systems based on the disruption of proteins and membranes. Furthermore, it exists in two optically active forms, (+)-gossypol and (−)-gossypol, with the (−)-enantiomer being more reactive toward biological systems, notably nonruminants (Celorio-Mancera Mde et al., 2011; Rathore et al., 2020).

High levels of free gossypol can cause acute clinical symptoms, including respiratory problems, anorexia, reduced weight gain, apathy, weakened immunity, and potentially death within days (Gadelha et al., 2014). In a study on sheep, lambs born to mothers who consumed a diet containing cottonseed exhibited noticeably stunted growth and lower testis weight relative to their overall body weight. Additionally, testosterone levels were reduced. Maternal exposure to gossypol has the potential to disrupt male reproductive function in offspring, leading to long-lasting or lifelong negative effects (Louvandini et al., 2020) It negatively affects reproductive processes in both females and males. In females, it can induce mitochondrial dysfunction, cause irregular estrous cycles, lower estradiol levels, and reduce ovarian follicles, ultimately resulting in lower pregnancy rates (Ding et al., 2021; Luz et al., 2018). It impairs oocyte maturation, disrupts mitochondrial function, and induces oxidative stress. It also reduces sperm production and impairs sperm motility. Females exposed to gossypol face issues with the estrous cycle, pregnancy, and early embryonic development (Ding et al., 2021). It also has negative effects on male reproductive health, including reduced sperm count, motility, and testosterone concentration, as well as increased abnormalities in sperm. It can also impact the mineral content of the testis and sperm production (El-Sharaky et al., 2010; Louvandini et al., 2020).

Gossypol poisoning has been linked to a decrease in antioxidants in the body’s tissues and induced oxidative stress via an elevated level of reactive oxygen species (ROS), leading to inflammation and mitochondrial dysfunction (Ding et al., 2021; Saleh et al., 2018). In high amounts, it negatively affects the production of energy in the body by interfering with the activity of enzymes involved in the mitochondrial electron transport chain and oxidative phosphorylation, which are responsible for generating energy. Additionally, it has an impact on the heart. It reduces the force with which the heart contracts and limits the extent to which the cardiac fibers can contract (Gadelha et al., 2014). Its poisoning is a condition that has been observed in various animals, such as chickens, sheep, dogs, pigs, and goats. Compared with ruminants, nonruminant animals such as birds, pigs, fish, and rodents are more prone to gossypol toxicity (Gadelha et al., 2014). Previous studies of gossypol toxicity in specific tissues of different animals, such as the ovarian follicles of sheep (Câmara et al., 2015), mouse macrophages (Cao et al., 2023), the testicular and hepatic tissues of male rats (El-Sharaky et al., 2010), northern bob whites (Farthing et al., 2019), acute hepatotoxicity in rats (Fonseca et al., 2013), gossypol enantiomers in broilers (Lordelo et al., 2005), laying and broiler breeder hens (Lordelo et al., 2007), goat erythrocyte membrane osmotic fragility (Matondi et al., 2007), mitochondrial dysfunction and oxidative stress in mice (Ding et al., 2021) and reproductive damage and the role of vitamin E in rats (Santana et al., 2015), have been reported. However, studies on the toxicity of gossypol on the hematology and histopathology of mice (female and male) are still lacking. The current study aims to examine the mechanisms of acute toxicity in blood and histological tissues (kidney, liver, lung, and spleen) as well as reproductive tissues from both male and female mice. The objective of this study was to explore gossypol toxicity and its effects on the expression of genes involved in reproduction and the liver in mouse tissues. The findings from this study can be utilized to assess the safety of gossypol as a potential option for food and feed.

Transcriptome analysis of mouse tissues has been extensively studied via various techniques (West et al., 2016). The mouse has been widely used as a model organism for studying human diseases and for evaluating drug safety and efficacy. Many diseases and drug effects exhibit tissue specificity that may be reflected by tissue-specific gene expression profiles (Uhl and Warner, 2015). RNA-seq has been employed to create a comprehensive BodyMap across 17 tissues, revealing tissue-specific gene expression patterns and identifying housekeeping genes (Li et al., 2022). Gossypol disrupts spermatogenesis and steroidogenesis in male mice by reducing cell viability, the mitochondrial membrane potential, and the expression of testis development-related genes while modulating the *MAPK* and *PI3K* signaling pathways (Lim et al., 2019). In mice, short-term gossypol exposure causes reversible reproductive toxicity and nephrotoxicity, affecting testicular and kidney morphology (Wang et al., 2023). Investigating the effects of gossypol on mouse health, particularly fertility, and identifying response genes is crucial for optimizing its application in feed and food. This study addresses the gap in comprehensive research by examining gossypol toxicity in mouse tissues and identifying key genes involved in the fertility response.

## 2 Materials and methods

### 2.1 Experimental animals

To determine the effects of gossypol on health, we used mice as model animals for toxicity tests.

The mice used in this study were all obtained from the Laboratory Animal Center of Nantong University. Five mice from each cage (DMSO, 20 mg/kg, 40 mg/kg and 80 mg/kg) from both sex which means 20 males and 20 females each with total n = 40 mice for the hematology and histopathology experiment were housed and kept at a constant temperature (22 °C ± 1 °C) and humidity environment with a 12-h light/dark cycle. All the mice were allowed free access to food and water during the experimental procedure. For this experiment, C57BL/6 mice aged 5 weeks and weighing between 15 and 20 g and approximately 4–5 weeks were selected, provided by the Experimental Animal Center of Nantong University (Sun et al., 2012). During the experiment, the animals were provided sufficient water and food at a room temperature of 25 °C, and the environment in which the mice were exposed was maintained under a 12-h light/dark cycle. Male and female mice were randomly assigned to four groups, with five mice per cage. The powder form of (+)-gossypol from cotton seeds was used for the experiment and was dissolved in a dimethyl sulfoxide (DMSO) solution. Pre-trial experiment was done on the dose calculation. 40 mg/kg from the pre-trial showed significant variation with the control group from the and make it half (20 mg/kg) and double (80 mg/kg) for the final experiment. Then we take the medium rate (40 mg/kg) from the pre-trial and make it half (20 mg/kg) and double (80 mg/kg) for the final experiment. The control group was pure DMSO; the second group (20 mg/kg) contained 8 mg/mL of the original solution of gossypol in DMSO to 20 mg/kg; the third group (40 mg/kg) contained 8 mg/mL of the original solution of gossypol in DMSO to 40 mg/kg; and the fourth group (80 mg/kg) contained 8 mg/mL of the original solution of gossypol in DMSO to obtain 80 mg/kg gossypol. The drug was administered by oral gavage for two consecutive weeks at a fixed time according to the weight of the mice.

The mouse experiments were conducted according to the Guide for the Care and Use of Laboratory Animals, and the protocols were approved by the Institutional Animal Care and Use Committee of Nantong University (Approval No. IACUC20241220-018). In accordance with the Animal Research: Reporting of *In Vivo* Experiments (ARRIVE) guidelines, we prepared the animal experiments and reported them. All animal experiments were conducted in accordance with the Nursing and Use Guidelines for Experimental Animals of the Key Laboratory of Nantong University and Neuro-regeneration, as well as the Nursing and Use Guidelines for Experimental Animals of the National Institutes of Health in the United States. The Animal Care and Use Committee of Nantong University approved the protocol used in this study.

### 2.2 Clinical observations and biological sampling

Brief veterinary examinations will be conducted at the beginning and end of the study. Observations will be made twice daily throughout the study, with a focus on general health, mortality, and physical condition (injury), and weekly measurements will be taken on days 0, 7, and 14 for body weight and feed consumption (Pitchford et al., 2018). By injecting compound anesthetics (10 mg/kg xylazine, 95 mg/kg ketamine, and 0.7 mg/kg acepromazine) into the abdominal cavity of the mice, after deep anesthesia, the limbs of the mice were fixed, the abdomen facing upward was lifted, the abdomen was wiped with 75% alcohol, the chest skin and muscles of the mice were cut open, the mouse heart was completely exposed, and cardiac blood collection was performed. Next, the blunt surgical needle was inserted into the left ventricle, which was cut through the right atrial appendage, and the whole body was infused with 0.9% physiological saline. After the capillaries in the intestinal area changed from red to white, they were replaced with 4% paraformaldehyde. After the mouse limbs became stiff, the mouse liver, spleen, lungs, kidneys, ovaries, and testes were quickly removed. Mouse tissues from the control group and treated group will be fixed in 4% paraformaldehyde at 4 °C until further steps. The samples will be embedded in paraffin, sectioned, and stained with hematoxylin and eosin (Cardiff et al., 2014; Li et al., 2018).

### 2.3 Hematological and histopathological parameters

At the end of the experiment, blood samples were drawn from each animal via cardiac puncture. The blood was placed in an anticoagulant tube, which was quickly inverted to prevent blood clotting. A routine blood analyzer was used to determine hematological parameters, such as white blood cell count (WBC), red blood cell count (RBC), hemoglobin (HGB), hematocrit (HCT), mean cell volume (MCV), mean corpuscular hemoglobin concentration (MCHC), mean corpuscular hemoglobin (MCH), and platelet count (PLT), automatically via the BC-2800 VET/Mindray device. Differential counting will be performed via a smear of blood stained by the panoptic (Brígido et al., 2021).

After euthanasia, ovary, testis, liver, kidney, lung, and spleen samples (1 cm thick) will be collected for histopathological examination. The tissues were placed in a centrifuge tube containing 4% paraformaldehyde, fixed at 4 °C for 24 h, and then placed in 10%, 20%, or 30% sucrose solution for gradient dehydration. After the tissue settled at the bottom of the centrifuge tube, the samples were dehydrated to the next concentration. After tissue dehydration was completed, the tissue was embedded in optimal cutting temperature (OCT) compound and stored at −80 °C. For slicing, the embedded tissue was embedded at a thickness of 12 μm, stained with hematoxylin and eosin (HE), and observed under a regular optical microscope (100X). Slides will be evaluated by an independent, certified histopathologist, and the results will be confirmed by a second independent, certified histopathologist (Brígido et al., 2021).

### 2.4 Transcriptome analysis

In the initial study, the results revealed that routine blood and histopathology results were adversely affected in the mice treated with gossypol, particularly those in the 80 mg/kg gossypol group, compared with those in the control group. This was apparent through a significant increase in neutrophil granulocytes and monocytes, as well as a notable decrease in lymphocytes. Furthermore, gossypol treatment resulted in decreased spermatogenesis, follicular degeneration in the ovary, and tissue damage to the liver, kidney, lung, and spleen. As a result, we decided to continue the study to evaluate its molecular effects via RNA sequencing. For the transcriptome study, we organized two treatment groups, namely, the control (DMSO) group and the 80 mg/kg gossypol group, with both male and female categories. The trial used mice aged 10–12 weeks to observe reproductive and liver tissue damage. The gossypol solution was administered for 2 weeks, after which tissue samples from the liver, ovary and testis were taken for transcriptome sequencing in three biological replicates. A total of 18 samples were taken, promptly submerged in liquid nitrogen and stored at −80 °C (Yao et al., 2023).

### 2.5 cDNA library construction and sequencing

Total RNA was extracted via a TRIzol reagent kit (Invitrogen, Carlsbad, CA, USA) according to the manufacturer’s protocol. RNA quality was assessed on an Agilent 2100 Bioanalyzer (Agilent Technologies, Palo Alto, CA, USA) and checked via RNase-free agarose gel electrophoresis (Meyers et al., 1976). After total RNA was extracted, eukaryotic mRNA was enriched with oligo(dT) beads (Green and Sambrook, 2019). mRNA was reverse transcribed into cDNA via the NEBNext Ultra RNA Library Prep Kit for Illumina (NEB 7530, New England Biolabs, Ipswich, MA, USA). The resulting cDNA library was sequenced via Illumina NovaSeq-6000 by Gene *Denovo* Biotechnology Co. (Guangzhou, China).

### 2.6 Differentially expressed genes and principal component analysis

RNA differential expression analysis was performed via DESeq2 software between two different groups (and via edgeR between two samples) (Li et al., 2022). The genes/transcripts with a false discovery rate (FDR) below 0.05 and an absolute fold change ≥2 was considered differentially expressed genes/transcripts. Principal component analysis (PCA) was performed with the R package gmodels (http://www.r-project.org/) in this experiment. PCA is a statistical procedure that converts hundreds of thousands of correlated variables (gene expression) into a set of values of linearly uncorrelated variables called principal components. PCA is largely used to reveal the structure/relationship of samples (Jolliffe and Cadima, 2016).

### 2.7 WGCNA and coexpression network construction

Weighted gene coexpression network analysis (WGCNA) for blood analysis and transcriptome analysis of tissues from mice served as the phenotype and genotype data, respectively. A correlation-based association between the gene modules and the different tissue samples from the mice was conducted following the default parameters set within the WGCNA framework. Once the network was established, transcripts with similar expression patterns were combined into a single module, and eigengenes for these modules were identified (Langfelder and Horvath, 2008). Cytoscape software (version 3.7.2) was used to analyze the coexpression networks of four modules, blue, brown, gray, and turquoise, which were strongly positively correlated with mouse blood analysis (Shannon et al., 2003). The top 20 genes from the blue, brown, gray and turquoise modules out of 4,839, 1,207, 33 and 6,613 genes, respectively, were imported into Cytoscape on the basis of their correlation values. Our approach involved the use of CytoHubba, a Cytoscape extension, to identify hub genes and visualize the network. The degree of the topological coefficients connected with each node of the 20 genes was used to select hub/key genes from each section/subsection of the treatment and different tissues. The hub genes in the network were then determined on the basis of the largest number of positive correlations among the nodes.

### 2.8 GO term, KEGG pathway and reactome enrichment analyses

Using filtered DEGs, Gene Ontology (GO), Kyoto Encyclopedia of Genes and Genomes (KEGG) and Reactome enrichment analyses were performed. The gene functional classification system categorizes GO into three ontologies: molecular function, cellular component and biological process (Ashburner et al., 2000). KEGG pathway enrichment analysis revealed significantly enriched metabolic pathways or signal transduction pathways in DEGs compared with the whole-genome background (Kanehisa and Goto, 2000). Similarly, reactome biological pathways focus on reactions as the core unit within the reactome data model. Reactome enrichment analysis revealed significantly enriched reactions in DEGs compared with the whole-genome background. The calculated p-values were subjected to FDR correction, with FDR ≤ 0.05 used as a threshold (Fabregat et al., 2017).

### 2.9 Quantitative real-time PCR analysis and cell imaging

To confirm the sequencing results, we examined 15 genes from the ovary, 10 genes from the liver, and five genes from the testis via real-time quantitative PCR. We used first-strand cDNA with an M5 First Strand cDNA Synthesis Kit (Mei5bio, Beijing, China). The PCR process involved incubation at 50 °C for 50 min, 85 °C for 5 min, and then the cDNA was used as a template for RT‒qPCR. The GAPDH gene was used as a housekeeping gene (Barber et al., 2005). The NCBI database was used to design primers for the target genes (Supplementary Table S1). RT–qPCR was conducted via the ChamQ SYBR qPCR Master Mix Kit (Vazyme, Nanjing, China) on an ABI 7500 System (ABI, USA). The 20 μL RT–qPCR mixture included 10 μL of 2× ChamQ SYBR qPCR Master Mix, 0.4 μL of each forward and reverse primer, 1 μL of the cDNA template and 8.2 μL of ddH_2_O. The procedure included predenaturation at 95 °C for 30 s, 95 °C for 15 s, 60 °C for 30 s, a melting curve at 95 °C for 15 s, 60 °C for 60 s, and 95 °C for 15 s. The 2^−ΔΔCT^ method was used to calculate the relative expression levels of the genes (Schmittgen and Livak, 2008). Three technical and biological replications were performed. Significant differences were illustrated through GraphPad Prism software (version 9.0.0). Analysis was performed via a t-test, and the results are displayed as the means ± standard deviations. The heatmap was generated via TBtools-II v2.012 (Chen et al., 2023).

### 2.10 Statistical analysis

Body weight and hematology data were analyzed via analysis of variance (ANOVA) via the SAS computer package version 9.2 at P < 0.05. When a notable difference among the treatment means was observed, Tukey’s studentized range (HSD) test was employed to compare the mean separation at P < 0.05 (Abdi and Williams, 2010). Data from each measurement and for each sex were analyzed separately. As the genders for each treatment group were housed separately, gender replication was maintained in the trial’s treatments. Three biological replicates were used during the data collection. The analyzed variables are presented as the means ± standard deviations.

## 3 Results

### 3.1 Effect of gossypol 20 mg/kg, 40 mg/kg and 80 mg/kg on body weight and mortality

The daily administration of gossypol via oral gavage for 14 days resulted in toxicity symptoms in the mice. In both the 40 mg/kg and 80 mg/kg groups, one male and two female mice died during the study period. Toxicity indicators were observed in the blood and histopathological tissues of the mice. The body weights of the mice were measured at the beginning (day 0), as well as on the 7th and 14th days of the study. No significant alterations in body weight were observed except in the 80 mg/kg group of female mice (Supplementary Table S2).

### 3.2 Effect of gossypol 20 mg/kg, 40 mg/kg and 80 mg/kg on hematology

Hematological analysis revealed statistically significant differences in only the percentages of neutrophil granulocytes, monocytes and lymphocytes in the male mouse group receiving gossypol compared with those in the DMSO group (Supplementary Table S3). The number of granulocytes significantly differed because the degree of damage to hematologic cells (neutrophils) was greater. High neutrophil counts indicate that the body is under stress. Infection, inflammation, stress, and vigorous exercise can increase neutrophil levels (neutrophilia). Similarly, there was a greater number of monocytes, indicating that damage was greater in the 80 mg/kg group because monocytes are a type of white blood cell that helps fight infections in the body. A high monocyte level may indicate inflammation, infection, blood disorders, and other health issues. There was a low level of lymphocytes in the 80 mg/kg group, which even decreased as the dose increased. Lymphocytopenia is the condition of having an abnormally low level of lymphocytes in the blood. Lymphocytes are white blood cells with important functions in the immune system.

Similarly, in the female mouse group, the hematological analysis revealed significant differences in neutrophil granulocytes, monocytes, the lymphocyte percentage, the number of neutrophils, the RBC count, the MCHC, the RDW-SD, and the P-LCR between the gossypol-treated group and the DMSO (control) group (Supplementary Table S4). High neutrophil granulocytes indicate stress on the body, which can be caused by infection, inflammation, stress, or vigorous exercise. The 80 mg/kg group presented increased monocyte levels, indicating potential inflammation, infection, or other health issues. Additionally, the lymphocyte percentage was low in the 80 mg/kg group, which indicates lymphocytopenia, a condition characterized by abnormally low levels of lymphocytes in the blood. Lymphocytes are a type of white blood cell that play a crucial role in the immune system. Red blood cell indices, such as the MCHC, RDW-SD, and P-LCR, are measured during blood count determination to quantify various conditions. An increase in RDW-SD indicates significant anisocytosis, differences in the evaluation of anemia, red cell inclusions, and membrane abnormalities on the basis of morphology in the groups. A low MCHC indicates gossypol poisoning in the red blood cells of the treated mice. The P-LCR significantly decreased in the groups due to thrombocytosis, an increased number of platelets in the blood, preventing normal blood flow.

### 3.3 Effect of gossypol 20 mg/kg, 40 mg/kg and 80 mg/kg on testicular histological architecture

Histopathological examination of the experimental mice surviving the oral gavage treatment of repeated doses of DMSO solution and cotton gossypol powder dissolved in DMSO at doses of 20 mg/kg, 40 mg/kg and 80 mg/kg for 14 days revealed relevant changes in tissue morphology both in the control group and in those treated with the samples. The testes were observed, and the morphology of the samples in the DMSO group was normal. However, various degrees of damage, including seminiferous tubule degeneration, interstitial edema, necrosis, atresion of Sertoli cells, thin walls of tubules, and congestion, were observed in the groups treated with 20 mg/kg, 40 mg/kg, and 80 mg/kg HWE (Supplementary Figures S1A–D). This damage results in depressed spermatogenesis, Sertoli cell toxicity, and degeneration of the seminiferous tubules. Similarly, the ovaries in the DMSO group appeared normal. In the 20 mg/kg group, there were signs of red blood cell infiltration, necrotic and atretic follicles, and an abnormal corpus luteum. The 40 mg/kg group presented an enlarged ovary with graphian and atretic follicles, as well as an abnormal corpus luteum. The 80 mg/kg group exhibited complete atresia of follicles, congested blood vessels, and degeneration of the corpus albicans of the corpus luteum (Supplementary Figures S1A–D). These findings revealed the toxicity of gossypol to female reproductive tissues.

The exposure of the mice to gossypol solution via oval gavage caused significant damage to the histology of the kidney. The male and female kidney observations revealed normal morphology in the control group treated with DMSO, whereas the 20 mg/kg, 40 mg/kg and 80 mg/kg groups presented kidney tissue damage. The doses of gossypol resulted in thickened walls of tubules, necrotic areas, and ruptured tubules in male kidneys. Similarly, hyperplasia of the white pulb, undefined demarcation between the red and white pulbs and degeneration/necrosis were observed in the female kidney group (Supplementary Figure S2).

In the control group of male and female mice, normal morphology was observed in the liver tissue, whereas the treated mice presented liver abnormalities. The 20 mg/kg and 40 mg/kg groups presented hyperemia near the centrilobular vein and proximity to the central vein, as well as hepatocyte degeneration and necrosis. The 80 mg/kg group exhibited hemorrhage, an enlarged centrilobular vein, expanded interlobular space (sinusoidal spaces, sinusoids), inflammatory infiltration inside blood vessels, disorganization of the hepatic parenchyma, and tissue calcification. Moreover, observations of female mice treated with 20 mg/kg and 40 mg/kg CBZ revealed infiltration of Kupffer cells, narrowing of the intralobular space, and congestion around the central vein and portal trade. In the last group, which was treated with 80 mg/kg, a disorganized configuration with no or narrow demarcation between hepatocyte lobules was observed (Supplementary Figure S3).

In the spleen, both the male and female groups exhibited normal morphology after DMSO administration. The red pulp contained red blood cells, whereas the white pulp consisted of lymphocytes. However, the treated male mice displayed red blood cell infiltration, lymphocyte infiltration, necrosis, distorted red pulp, and unclear boundaries. On the other hand, the treated female mice presented hyperplasia of white pulp, undefined demarcation between red and white pulp, and degeneration/necrosis of red pulp (Supplementary Figure S4).

**FIGURE 1:**
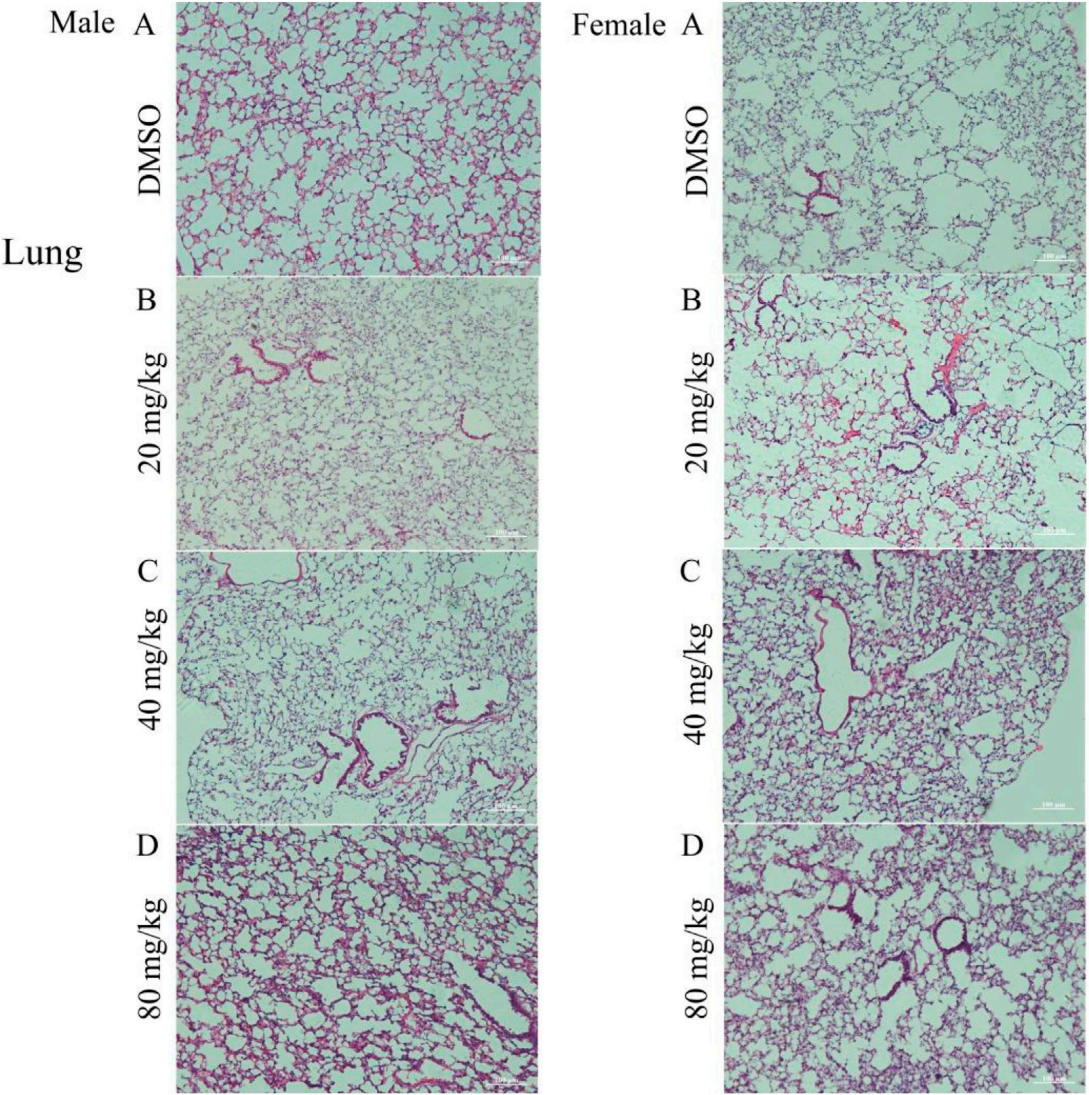
Photomicrograph of the lungs of mice treated with DMSO or gossypol solution of cottonseed powder. **(A)** group treated with DMSO (control group); **(B)** group treated with 20 mg/kg gossypol; **(C)** group treated with 40 mg/kg gossypol; **(D)** group treated with 80 mg/kg gossypol. N.B., Right-side images represent male mice, and the left-side images represent female mice, Magnification = ×10 and scale bar = 100 µm were used for all images in Photomicrograph.

Histopathological analysis of lung tissue sections from the test groups revealed a normal morphological structure in the control group, whereas the treated group exhibited pathological alterations. The treated male group exhibited collapsed alveolar sacks, alveolar fibrosis, histocyte infiltration, thickened alveolar walls, collapse of sacks and fibrosis. Similarly, in female mice, lung observations after treatment revealed various abnormalities, including hyperemic alveolar ducts, intra-alveolar hemorrhage, and infiltration of alveolar dust cells and macrophages. Other findings included narrowing of alveolar sacks, pulmonary congestion, and alveolar edema (Figure 1).

### 3.4 Mouse transcriptome profiling

After transcriptome analysis of the mouse tissues, 18 cDNA libraries were constructed. A total of 113.6 Gb of raw reads were obtained from these libraries. After filtering via fastp, 112.4 GB of clean reads were identified, with high-quality reads, and 21,899 genes were identified. The filtered reads were aligned to the reference genome via the upgraded version of HISAT2 software, and the other parameters were set to the defaults. Data curation was performed with the Q20 and Q30 percentages of BF and AF, respectively, and the sequencing quality was >98% and >96%, respectively, in both categories (Table 1).

**TABLE 1:** Summary of the transcriptome analysis results.

Sample	Raw data	Q20 (BF)	Q30 (BF)	Clean data	Q20 (AF)	Q30 (AF)
DL-1	6150245100	98.87%	96.87%	6085399123	99.05%	97.17%
DL-2	6583172400	98.86%	96.86%	6513711898	99.05%	97.17%
DL-3	6198962700	99.00%	97.09%	6145061328	99.14%	97.32%
GL-1	6196878300	98.87%	96.77%	6132400581	99.04%	97.07%
GL-2	5652924600	98.95%	96.96%	5594646720	99.11%	97.23%
GL-3	6406365000	98.91%	96.92%	6343376772	99.09%	97.22%
DO-1	6291696000	98.59%	96.26%	6207931494	98.84%	96.67%
DO-2	6418887300	98.58%	96.24%	6337349856	98.84%	96.65%
DO-3	7021831200	98.62%	96.36%	6940396456	98.86%	96.75%
GO-1	5872023600	98.04%	94.63%	5784180335	98.30%	95.04%
GO-2	6251792700	98.58%	96.25%	6179431800	98.83%	96.64%
GO-3	6433111500	98.74%	96.64%	6361463929	98.96%	97.00%
DT-1	6887275200	98.77%	96.66%	6813415810	98.99%	97.02%
DT-2	6107950800	98.82%	96.71%	6042040378	99.01%	97.02%
DT-3	6365737200	98.82%	96.71%	6295900943	99.02%	97.04%
GT-1	6474910200	98.88%	96.84%	6408061472	99.06%	97.14%
GT-2	6860489700	98.83%	96.71%	6795029389	99.02%	97.03%
GT-3	5474699400	98.70%	96.33%	5418243166	98.92%	96.68%
Total	113.6 Gb	>98%	>96%	112.4 Gb	>98%	>96%

Note: DL - DMSO, liver; GL, gossypol liver; DO - DMSO, ovary; GO, gossypol ovary; DT - DMSO, testis; GT, gossypol testis mouse tissues used for the transcriptome analysis. 1, 2, and three represent three biological replications.

### 3.5 Differentially expressed genes (DEGs)

**FIGURE 2:**
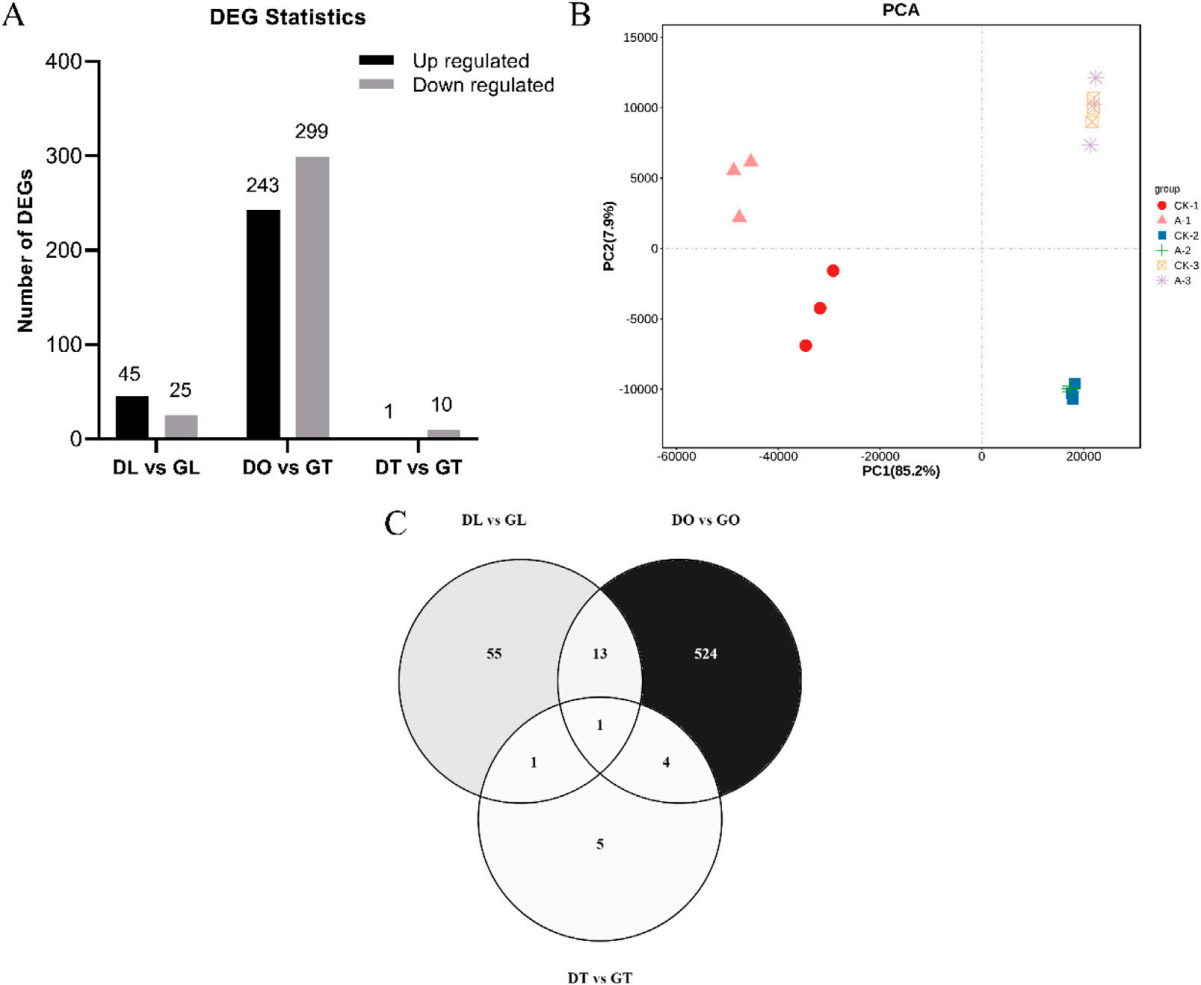
The sum of differentially expressed genes (DEGs) identified via RNA-Seq data for the mouse tissue samples. **(A)** Number of upregulated and downregulated DEGs identified in the three comparison groups. **(B)** Principal component analysis of 18 mouse tissue samples (DL vs. GL, DO vs. GO and DT vs. GT) from three biological replicates. **(C)** Gene distributions among different tissue samples (DL vs. GL, DO vs. GO and DT vs. GT) from DEGs.

The DEG analysis revealed that 289 genes were upregulated and that 334 genes were downregulated. Specifically, 45 genes whose expression was upregulated and 25 whose expression was downregulated in the DL vs. GL groups, 243 whose expression was upregulated and 299 whose expression was downregulated in the DO vs. GO groups, and 1 whose expression was upregulated and 10 whose expression was downregulated in the DT vs. GT groups were identified (Figure 2A). To further confirm the relationships among the three comparison groups, principal component analysis (PCA) was performed on the expressed genes. The results indicated that the genes expressed in the same group in the three groups could be aggregated, except for a small deviation in the DL vs. GL groups (Figure 2B). The gene distribution in the DEGs among different tissue samples varied from 524 in DO vs GO, 55 in DL vs GL, 5 in DT vs GT with a single gene as a common in all groups (Figure 2C). The similar gene expression patterns in the comparison groups indirectly proved the reliability of the transcriptome data.

### 3.6 Functional enrichment analysis of DEGs

**FIGURE 3:**
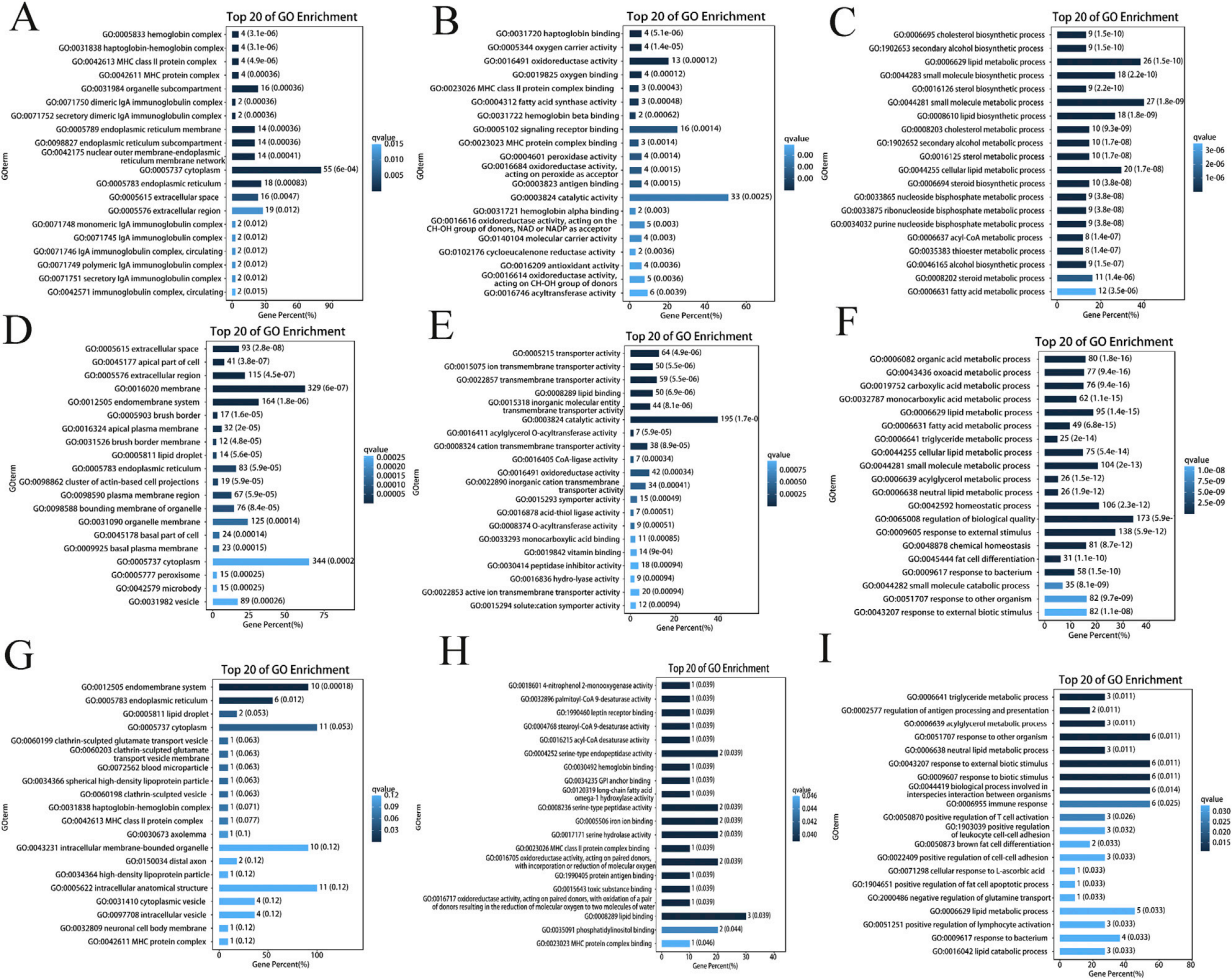
Gene ontology annotation analysis. **(A)** DEG annotations in the cellular components of the ovary; **(B)** DEG annotations in the molecular functions of the ovary; **(C)** DEG annotations in the biological processing categories of the ovary; **(D)** DEG annotations in the cellular components of the liver; **(E)** DEG annotations in the molecular functions of the liver; **(F)** DEG annotations in the biological processing categories of the liver; **(G)** DEG annotations in the cellular components of the ovary; **(H)** DEG annotations in the molecular functions of the ovary; **(I)** DEG annotations in the biological processing categories of the ovary.

To reveal the functions of the DEGs, Gene Ontology (GO) classification, Kyoto Encyclopedia of Genes and Genomes (KEGG) pathway annotation and reactome enrichment analysis were performed on the identified DEGs. According to the GO enrichment results, the top 20 pathways were selected for visualization from liver, ovary and testis tissues (Figure 3). Among these DEGs, the majority of genes were enriched within cellular components, with 344, 55 and 11 genes associated with the cytoplasm (GO:0005737) in the ovary, liver and testis, respectively. In terms of molecular function, 195 and 33 genes were enriched in catalytic activity (GO:0003824) in the ovary and liver groups, respectively, whereas three genes from testis tissue were enriched in lipid binding (GO:0008289) functions. Additionally, the genes enriched in the biological process category were predominantly associated with the regulation of biological quality (GO:0065008; 173 genes) in the ovary, small molecule metabolic process (GO:0044281; 27 genes), and lipid metabolic process (GO:0006629; 26 genes) in the liver. Furthermore, six genes were enriched in immune response (GO:0006955), biological process involved in interspecies interaction between organisms (GO:0044419), response to biotic stimulus (GO:0009607), response to external biotic stimulus (GO:0043207), and response to other organism (GO:0051707) biological process in testicular mice.

We also performed KEGG pathway enrichment analysis for the pathways linked to these target genes (Supplementary Figure S5). On the basis of the number of enriched genes in each pathway and their corresponding p values, we selected the top 20 enriched pathways. One hundred and 20 genes were enriched in pathways related to metabolic pathways in the ovary and liver, respectively. In the testis, two genes associated with each biological process were enriched in the PPAR signaling pathway, the AMPK signaling pathway, alcoholic liver disease and nonalcoholic fatty liver disease. Reactome enrichment analysis of the DEGs revealed 105 genes involved in metabolism (R-MMU-1430728) and 17 genes involved in the metabolism of lipids (R-MMU-556833) in both the ovary and liver. The top 20 results of the reactome enrichment analysis of testis tissue involved various biological pathways, such as CIDEC binding to lipid droplets (R-MMU-8856630), leptin binding to leptin receptor (R-MMU-2586558) and haptoglobin binding to hemoglobin (R-MMU-2168885) (Supplementary Figure S5). These results suggest that the pathways enriched by these genes may play crucial roles in the response to gossypol toxicity in mouse tissues.

### 3.7 WGCNA and coexpression network analysis

**FIGURE 4:**
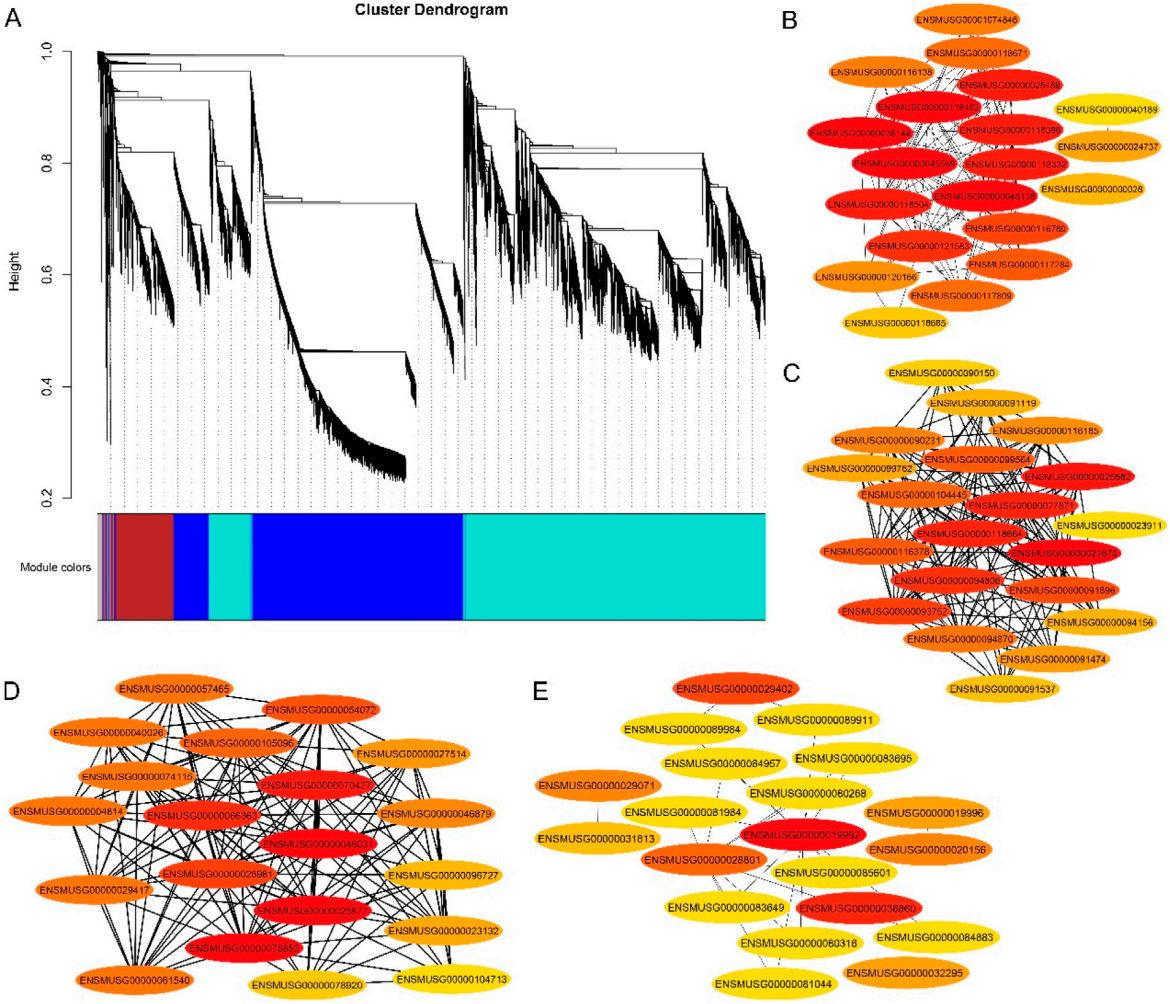
Gene networks and key candidate genes involved in gossypol toxicity identified by WGCNA. **(A)** Cluster dendrogram network construction from three mouse tissues; **(B)** Blue module with hub genes linked to mouse fertility; **(C)** Brown module with hub genes linked to mouse fertility; **(D)** Gray module with hub genes linked to mouse fertility; **(E)** turquoise module with hub genes linked to mouse fertility.

Weighted gene coexpression network analysis (WGCNA) was carried out utilizing the FPKM values of commonly expressed DEGs from the mouse tissues. On the basis of WGCNA, four significant modules, namely, blue, brown, gray and turquoise, were highly positively associated with mouse DMSO/gossypol blood content. The blue module contained 4,839 genes, the brown module contained 1,207 genes, the gray module contained 33 genes, and 6,613 genes were present in the turquoise module (Figure 4A). Four genes from the blue module *ENSMUSG00000048138* (*Dmrt2*), *ENSMUSG00000045569* (*Mc2r*), *ENSMUSG00000036144* (*Meox2*)*, ENSMUSG00000025488 (Cox8b)*, three genes from the brown module *ENSMUSG00000025582* (*Nptx1*), *ENSMUSG00000021670* (*Hmgcr*) and *ENSMUSG00000027871* (*Hsd3b1*), four genes from gray module *ENSMUSG00000070427* (*Il18 bp*), *ENSMUSG00000025877, ENSMUSG00000046031* and *ENSMUSG00000078853,* two genes in turquoise module *ENSMUSG00000019992* and *ENSMUSG00000036860* were identified as hub genes (Figures 4B–E).

### 3.8 RT‒qPCR expression analysis

**FIGURE 5:**
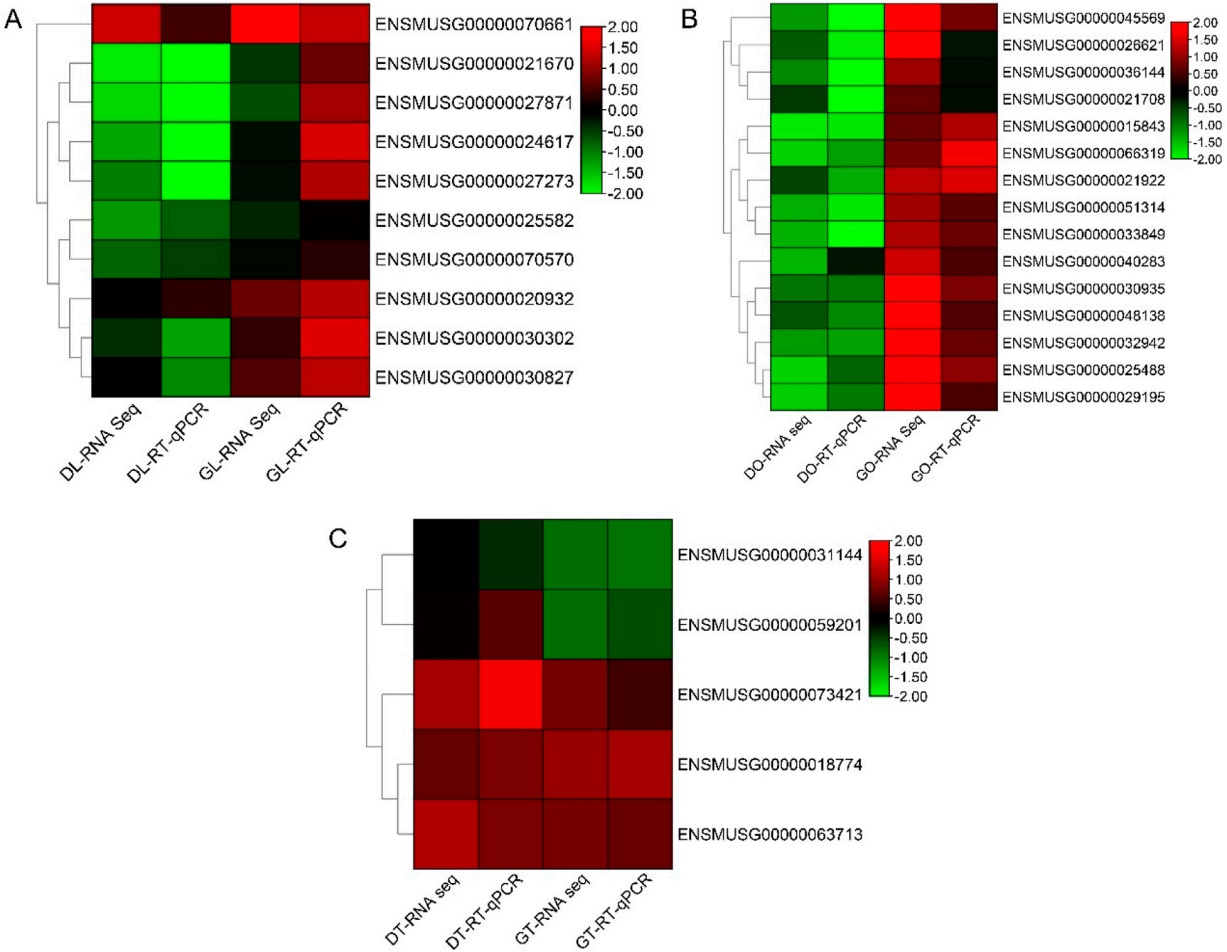
RT‒qPCR analysis of the hub genes. **(A)** Heatmap of log2-fold changes in the RNA-Seq (FPKM) and RT‒qPCR data of liver tissues. **(B)** Heatmap of log2-fold changes in the RNA-Seq (FPKM) and RT‒qPCR (RT‒qPCR) data of ovary tissues. **(C)** Heatmap of log2-fold changes in the RNA-Seq (FPKM) and RT‒qPCR (RT‒qPCR) data of the ovarian tissues. Note: DL, liver tissue treated with DMSO; GL, liver tissue treated with gossypol; DO, ovary tissue treated with DMSO; GO, ovary tissue treated with gossypol; DT, testis tissue treated with DMSO; GT, testis tissue treated with gossypol.

RT‒qPCR analysis was performed to validate the DEGs from the ovary, liver, and testis RNA-Seq data. The primers used for the analysis were designed via NCBI BLAST, and their sequences are provided in the supplementary table. Some genes in the three tissues were upregulated in the gossypol treatment group but downregulated in the DMSO treatment group. On the basis of the RNA-Seq and RT‒qPCR results, we identified the genes with the highest expression in gossypol-treated mice: *ENSMUSG00000025488* (*Cox8b*), *ENSMUSG00000048138* (*Dmrt2*), and *ENSMUSG00000045569* (*Mc2r*) in ovary tissue; *ENSMUSG00000021670* (*Hmgcr*) and *ENSMUSG00000027871* (*Hsd3b1*) in liver tissue; and *ENSMUSG00000018774* (*Cd68*) in testis tissue. These genes may be true candidates linked to fertility in mice. The correlations of the RNA-Seq and RT‒qPCR analyses were high and positive in all tissues (r = 0.77, r = 0.66, and r = 0.92 in the ovary, liver and testis, respectively) (Figure 5).

### 3.9 Immunofluorescence staining and expression profiling of the *DMRT2* gene

**FIGURE 6:**
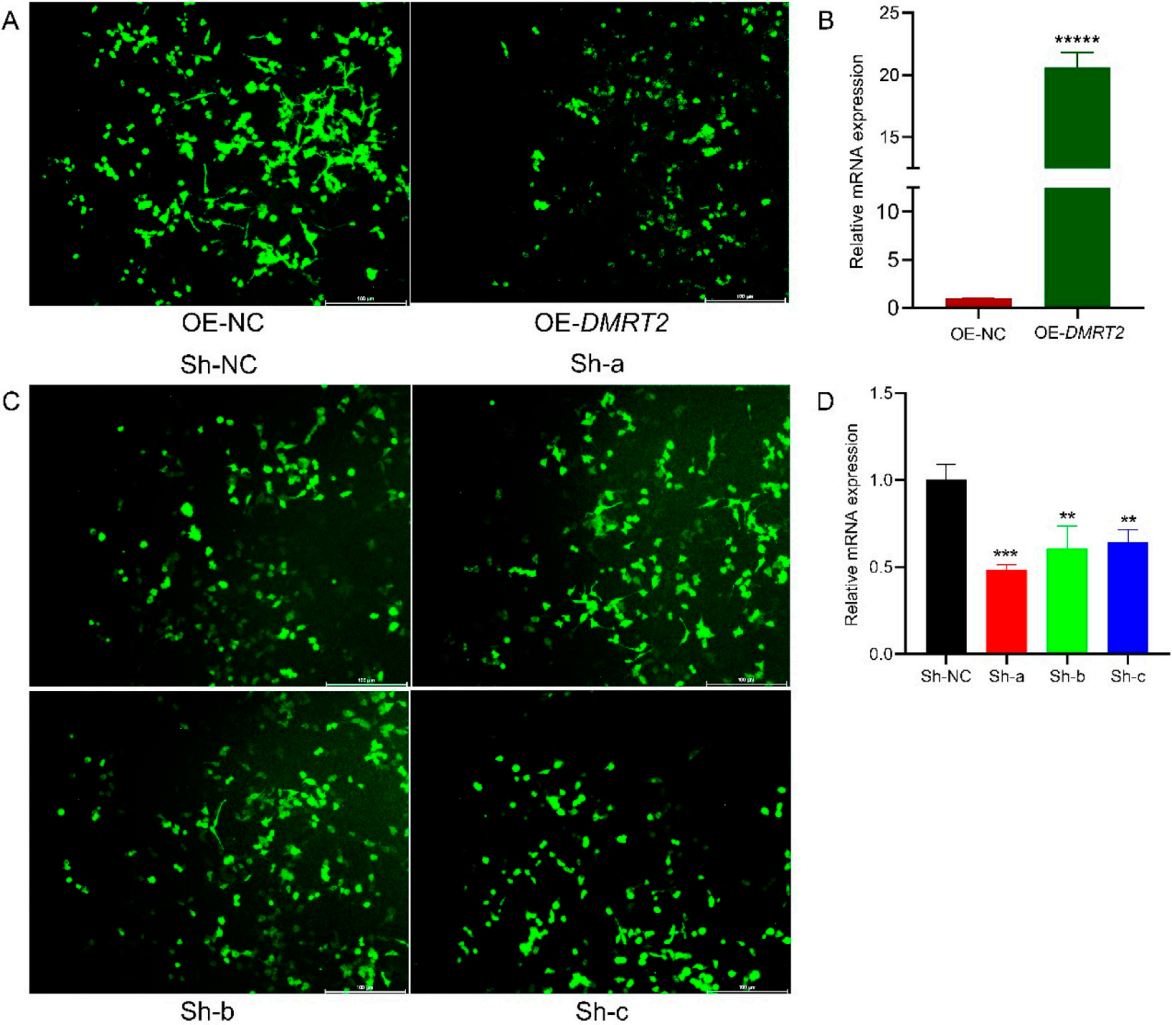
Immunofluorescence staining of the *DMRT2* gene. **(A)** Immunofluorescence staining image of Lv-*Dmrt2*-transfected cells. **(B)** Changes in the relative expression of *Dmrt2* mRNA after Lv-*Dmrt2* transfection. **(C)** Immunofluorescence staining image of Lv-shRNA-transfected cells. **(D)** Changes in the relative expression of *Dmrt2* mRNA after Lv-shRNA transfection. * Denotes a significant difference at P ≤ 0.05, ** indicates a significant difference at P ≤ 0.01, and *** indicates a significant difference at P ≤ 0.001.

To investigate the role of *Dmrt2* in cells, we overexpressed and knocked down the *Dmrt2* gene *in vitro* via the use of lentiviral particles. Microscopic examination confirmed successful lentiviral transfection. Immunofluorescence staining of OE-*Dmrt2* cells revealed a strong, filamentous network with heterogeneous intensity across cells, which was noticeably reduced in the OE-NC control (Figure 6A). Lv-*Dmrt2* (OE-*Dmrt2*) was used for overexpression, and Lv-shRNA (Sh-a, Sh-b, Sh-c) was used for knockdown. RT‒qPCR analysis confirmed significant OE-*Dmrt2* overexpression in the cells transfected with the Lv-Dmrt2 lentivirus compared with the OE-NC control cells (Figure 6B).

Similarly, the staining intensity varied, with Sh-NC cells exhibiting strong signals, whereas Sh-a, Sh-b, and Sh-c cells showing weaker signals. Compared with that in control cells, the number of stained cells was significantly lower, suggesting a decrease in proliferation (Figure 6C). The RT‒qPCR results revealed that, compared with that in the control group, the effect of the fragment in the Sh-a, Sh-b, and Sh-c groups was significant relative to that in the control group (Figure 6D).

## 4 Discussion

Cottonseed (*Gossypium* spp.) is a valuable feed source with high protein content. It is commonly used as a feed supplement and primary ration for various livestock. However, cottonseed also contains gossypol, a toxic phenolic compound produced by the pigment glands of cotton plants (Câmara et al., 2015; Farthing et al., 2019). It is a conventional drug that has been identified as a potential treatment option for COVID-19and a broad-spectrum inhibitor of the SARS-CoV-2 RdRp enzyme, which could help in targeting the virus’s RNA-dependent RNA polymerases (Wang et al., 2022). It also targets mouse double minute 2 (*MDM2*) and vascular endothelial growth factor (*VEGF*) molecules, which are involved in tumor progression and have anticancer effects on human breast cancer (Xiong et al., 2017). It is crucial for cotton plant protection; it offers both natural and triggered defenses against pests and diseases. Additionally, it has potential applications as an antibacterial, anticancer, and male contraceptive agent (Zhao et al., 2020). On the other hand, it is a poisonous terpenoid found in seed glands, making it unsuitable for human consumption or as feed for nonruminants (Rathore et al., 2020). The toxic effects of gossypol on different species have been reported in several studies, including on the structure and nutritional composition of egg yolk (Zhu et al., 2019); affecting the transcriptome and mineral content of the testis (Louvandini et al., 2020); inhibiting human *HSD3B1* and *CYP19A1* enzymes (Dong et al., 2016); interfering with the follicular maturation of rats, mice and goats (Luz et al., 2018); and disrupting embryo development in heifers (Villaseñor et al., 2008).

In this study, we investigated the hematological and histopathological alterations and transcriptome analysis that occur following the administration of gossypol solution derived from cottonseed powder in mice. The results of the hematological analysis indicated a marked increase in the percentage of neutrophil granulocytes, monocytes and RDW-SD and a reduction in the percentage of lymphocytes, RBCs, MCHC and P-LCR. Similar to chanoclavine toxicity tests in mice, this study found significant differences in neutrophil percentage, total protein, and albumin levels between female mice fed a control diet and those fed chanoclavine (Finch et al., 2019). The testicular histopathology of the treated mice impaired spermatogenesis, testicular damage, and Sertoli cell toxicity. Female mice displayed ovarian damage with follicular atresia and corpus albicans degeneration. Gossypol-fed sheep had fewer viable and more atretic ovarian follicles than controls (Câmara et al., 2015). Gossypol causes low selenium content, reduced spermatogenesis, and degeneration of seminiferous tubules in lambs and rats (El-Sharaky et al., 2010; Louvandini et al., 2020). Similarly, gossypol *in vitro* trial revealed a significant reduction in the number of viable ovarian follicles, particularly in the primary and transition follicles, while causing a substantial increase in the number of atretic follicles. These findings indicate that gossypol directly affects ovarian follicles in sheep, promoting atresia (Câmara et al., 2015). Significant alterations in testicular pathology have been reported due to the use of different toxic materials, such as cadmium toxicity in rats (Shojaeepour et al., 2021), tramadol abuse in albino mice (Elghait et al., 2022), cyclophosphamide in Prague–Dawley rats (Ceribaşi et al., 2010) and dichloroacetic acid in male rats (El Arem et al., 2017). Similarly, ovarian histological toxicity in mice and rats has been reported to include haloperidol- and clozapine-induced toxicity in albino rats (Khalaf et al., 2019), cyclophosphamide-induced toxicity in rats (Yener et al., 2013), bortezomib-induced toxicity (Ben-Aharon et al., 2010; Mutluay et al., 2022), carboplatin and paclitaxel toxicity (Ntemou et al., 2022), zinc oxide particle toxicity (Xu et al., 2022), and melamine and cyanuric acid toxicity in mice (Sun et al., 2016).

Histopathological observation of the liver revealed severe hepatocyte damage and necrosis, hyperemia, enlargement of the centrilobular vein and hemorrhage, in addition to abnormal localization of hepatocytic nuclei in the treated mice. In previous studies, high-dose application of gossypol in northern bob whites caused significant lesions in various tissues, with the pancreas and liver being the most affected. The liver shows prominent pigment accumulation in hepatocytes and Kupffer cells, leading to changes in hepatocyte size and nuclei. The exact nature of the gray–green pigment could not be determined with routine stains (Farthing et al., 2019). DMSO-treated mice showed no overt toxicity, but after 14 days, kidneys exhibited tubular and pulb abnormalities like thickening, necrosis, rupture, thinning, hyperplasia, unclear demarcation, and degeneration. Gossypol induced macrophage death and decreased protein content, alongside increased expression of anti-inflammatory TTP family genes and proinflammatory cytokine genes. It also affected genes related to glucose transport and insulin signaling, including those implicated in arthritis, diabetes, and obesity (Cao and Sethumadhavan, 2023). Histopathological analysis revealed significant lung damage in moxa smoke-exposed rats compared to controls, including alveolar collapse, fibrosis, cell infiltration, thickened walls, hyperemic ducts, hemorrhage, and edema. Pulmonary congestion, congested blood vessels, and inflammatory cell infiltration were also observed, with the most pronounced effects in deceased rats and at higher moxa smoke concentrations, which induced structural changes in alveolar cell (Xu et al., 2021).

In the spleen, treated mice presented high degrees of damage, with infiltration of red blood cells, necrosis and unclear boundaries, significant infiltration of lymphocytes, distorted red pulp, increased white pulp, unclear boundaries between red and white pulp, and degeneration or necrosis of red pulp. Gossypol toxicity in northern bob whites revealed that histological examination of the heart revealed minimal pericardial adipose tissue and serous atrophy in birds, likely due to hyporexia, anorexia, and weight loss. Serous atrophy suggests a sudden shift to a negative energy balance, possibly caused by gossypol toxicosis (Farthing et al., 2019). In our study, the results indicated that, compared with those in the control group, the blood parameters and histopathology were negatively impacted following the treatment of the mice with gossypol. This was evident through the significant increase in neutrophil granulocytes and monocytes, as well as the notable decrease in lymphocytes. Additionally, gossypol treatment led to decreased spermatogenesis, follicular degeneration in the ovary, and tissue damage in the liver, kidney, lung, and spleen. These findings collectively suggest the toxic effects of gossypol on mice. Overall, our study offers new insights into the toxicity of gossypol in mammals.

Transcriptome analysis of ovary, liver and testis tissues has proven valuable for gossypol toxicity testing and understanding histotoxic mechanisms in mice. Studies have revealed differential gene expression in response to various compounds. RNA sequencing of mouse tissues from ovaries, livers and testes subjected to DMSO or gossypol treatment resulted in the generation of 18 transcriptome libraries. A total of 21,899 genes were identified, of which 623 were DEGs (including TFs), 289 genes were upregulated and 334 genes were downregulated. Functional enrichment analysis revealed that cellular component genes were associated with the cytoplasm in the ovary, liver, and testis. Catalytic activity was enriched in the ovary and liver for molecular function, whereas lipid binding was enriched in the testis. Additionally, ovary-enriched genes mainly regulate biological quality, whereas liver-enriched genes are involved in small molecule and lipid metabolism. Testicular mice presented enrichment of genes related to the immune response and interspecies/biotic stimulus responses. Gene ontology analysis has revealed important insights into lipid metabolism and the immune response in mice. Exposure to toxicants such as DEHP, TCDD, and triclosan alters genes involved in peroxisomal function, carboxylic and lipid metabolism, xenobiotic stress responses, and immune responses, resulting in species-specific variations and the upregulation of genes related to lipid metabolism and oxidation (Boverhof et al., 2006; Huang et al., 2020; Regnault et al., 2016). Disrupted lipid homeostasis can lead to membrane instability, lipid accumulation, oxidative stress, and inflammation. Systems genetics has also identified plasma lipid signatures that predict hepatic lipid accumulation in mice and revealed proteins and genetic variants that influence lipid abundance (Boverhof et al., 2006; Parker et al., 2019).

The ovary and liver were enriched in metabolic pathways, whereas the testis was enriched in PPAR signaling, AMPK signaling, alcoholic liver disease, and nonalcoholic fatty liver disease. Reactome enrichment analysis of the DEGs revealed their involvement in metabolic pathways, specifically lipid metabolism, in both the ovary and liver. In the testis, enrichment analysis highlighted pathways such as CIDEC binding to lipid droplets, leptin binding to its receptor, and haptoglobin binding to hemoglobin. Reproductive organs are metabolically significant and stress sensitive. The testis, which is enriched in metabolic pathways such as the AMPK signaling pathway, utilizes this pathway to regulate glycolysis and steroidogenesis. Testicular AMPK expression, modulated by nutritional status, affects testosterone production via steroidogenic enzymes (Abdou et al., 2014; Taibi et al., 2017). The liver expresses the least complex transcriptome, that is, the smallest number of genes detected in the liver across tissues, whereas the testis and ovary harbor more complex transcriptomes than other tissues do (Li et al., 2017). WGCNA revealed that the blue, brown, gray, and turquoise modules were significantly linked to gossypol and mouse fertility. Subsequent coexpression network and RT‒qPCR analyses revealed *Dmrt2* as a hub gene associated with mouse fertility. Double-sex and mab-3-related transcription factor (*DMRT*) genes, which encode highly conserved transcription factors with a unique DM domain, are essential for mammalian sexual development and maintenance. These genes regulate development across metazoans and have long controlled sexual differentiation. In mice, *Dmrt* genes act sequentially from embryonic development onward to establish and maintain robust and sustainable spermatogenesis (Zhang and Zarkower, 2017). The *Dmrt2* gene regulates neuronal proliferation and differentiation in the mouse cingulate cortex with sex-specific effects, highlighting its importance in neuronal development (Bermejo-Santos et al., 2025). *DMRT* TFs contribute to sex-specific development. Specifically, *Dmrt2* regulates proliferation and neuronal development in the mouse *CgCx*. *Dmrt2* downregulation leads to premature cell cycle exit and reduced cell density, potentially explaining the vulnerability of male embryos (with relatively high *Dmrt2* levels) to *Dmrt2* depletion. It also governs migration, axonal targeting, and gene expression in deep-layer neurons (Bermejo-Santos et al., 2025). Studies have shown that gossypol, a cotton seed polyphenol, induces cell death in mouse macrophages, which is correlated with increased expression of inflammatory, glucose transport, and insulin signaling genes (Cao and Sethumadhavan, 2023). It significantly decreased cell viability and soluble protein but markedly increased TTP (6–20×) and *ZFP36L1/2/3* (26–69×) mRNA levels. The levels of proinflammatory cytokines (*TNF*, *COX2*, *GM-CSF*, *INFγ*, *and IL12b*) also increased (39--458x). Gossypol upregulated *GLUT1/3/4*, INSR, *AKT1*, *PIK3R1*, and *LEPR* mRNAs but not APP, suggesting the occurrence of macrophage death and altered glucose transport/insulin signaling via the inflammatory and TTP pathways (Cao and Sethumadhavan, 2023). It impairs testis function by reducing cell viability and the mitochondrial membrane potential, suppressing testis development genes, and modulating the MAPK and PI3K signaling pathways both *in vitro* and *in vivo* (Lim et al., 2019). A maternal cottonseed diet reduced lamb growth, testis weight, and testosterone levels. Transcriptome analysis revealed that this diet altered testicular gene expression, impacting testis development, sperm biology, and signaling pathways. Network analysis suggests that gossypol-induced disruption of spermatogenesis gene coexpression impairs coregulation (Louvandini et al., 2020).

There are diverse roles of *DMRT* genes in sexual differentiation and development. *Dmrt1*, another family member, is essential for testicular differentiation and maintenance of male somatic cell fate (Zarkower and Murphy, 2022). In the fetal ovary, *Dmrt1* activates *Stra8* expression, promoting oogenesis and proper meiotic prophase (Krentz et al., 2011). The *Dmrt1* gene maintains testis or ovary fates in adult mammals, and mutation of the Dmrt1 gene can significantly affect the gonadal phenotype (Huang et al., 2017). *Dmrt2* functions extending beyond gonadal tissues to the brain are newly discovered, and *Dmrt4* mutant mice exhibit polyovular follicles and some male‒male copulatory behaviors but are otherwise viable and fertile (Balciuniene et al., 2006). Identifying candidate genes associated with gossypol and mouse fertility can provide resources for using cottonseed as feed and food. Moreover, the contribution of *DMRT2* mutations to mouse fertility through *in vivo* experiments will be of significant interest for further investigations.

## 5 Conclusion

In summary, Gossypol induced significant morphological alterations in hematologic and reproductive tissues, as well as visceral organs, as evidenced by histopathological and hematological changes. These changes disrupt normal cellular and tissue functions. Compared with the DMSO group, hematological (neutrophil granulocytes, lymphocytes, and monocytes) and histopathological (liver, lung, spleen, ovary, testis, and kidney) assessments revealed numerous clinical changes, indicating toxicity in treated mice. On the other hand, transcriptome analysis revealed 623 DEGs, and WGCNA identified four significant modules. *Dmrt2* (*ENSMUSG00000048138*), a hub gene from the blue module, was further validated by RT-qPCR analysis and was strongly positively correlated with the RNA-seq results. Furthermore, GFP analysis revealed that *Dmrt2* silencing and overexpression in mouse tissues via lentiviral vectors (Lv-Sh-a, Sh-b, Sh-c and OE-*Dmrt2*, respectively) resulted in corresponding decreases and increases in gene expression compared with those of the controls. Relative expression analysis revealed statistically significant upregulation of the *Dmrt2* gene in the ovary tissue after gossypol stress treatment, indicating that gossypol stress increases *Dmrt2* expression in the ovary. The *Dmrt2* gene likely plays a significant role in regulating mouse fertility. Gossypol, a toxin found in cotton, adversely affects mice, impacting their pathology, histology, and hematology. This study suggests that *Dmrt2* protects reproductive tissues from gossypol damage, enhancing our understanding of gene regulation in terms of mouse fertility and emphasizing the importance of further research and food safety.

## Data Availability

The data presented in the study are deposited in the NCBI repository, accession number PRJNA1157533.
