# Supplementary material for: Histopathological examination and transcriptomic profiling reveal gossypol toxicity-responsive genes related to fertility in mice
Source: Front Pharmacol. 2025 Aug 29;16:1654299. doi: 10.3389/fphar.2025.1654299 (PMC12425930; doi:10.3389/fphar.2025.1654299)
Supplement: Supplementary file 1 [file Supplementaryfile1.docx]

**Supplementary Figure Legends**


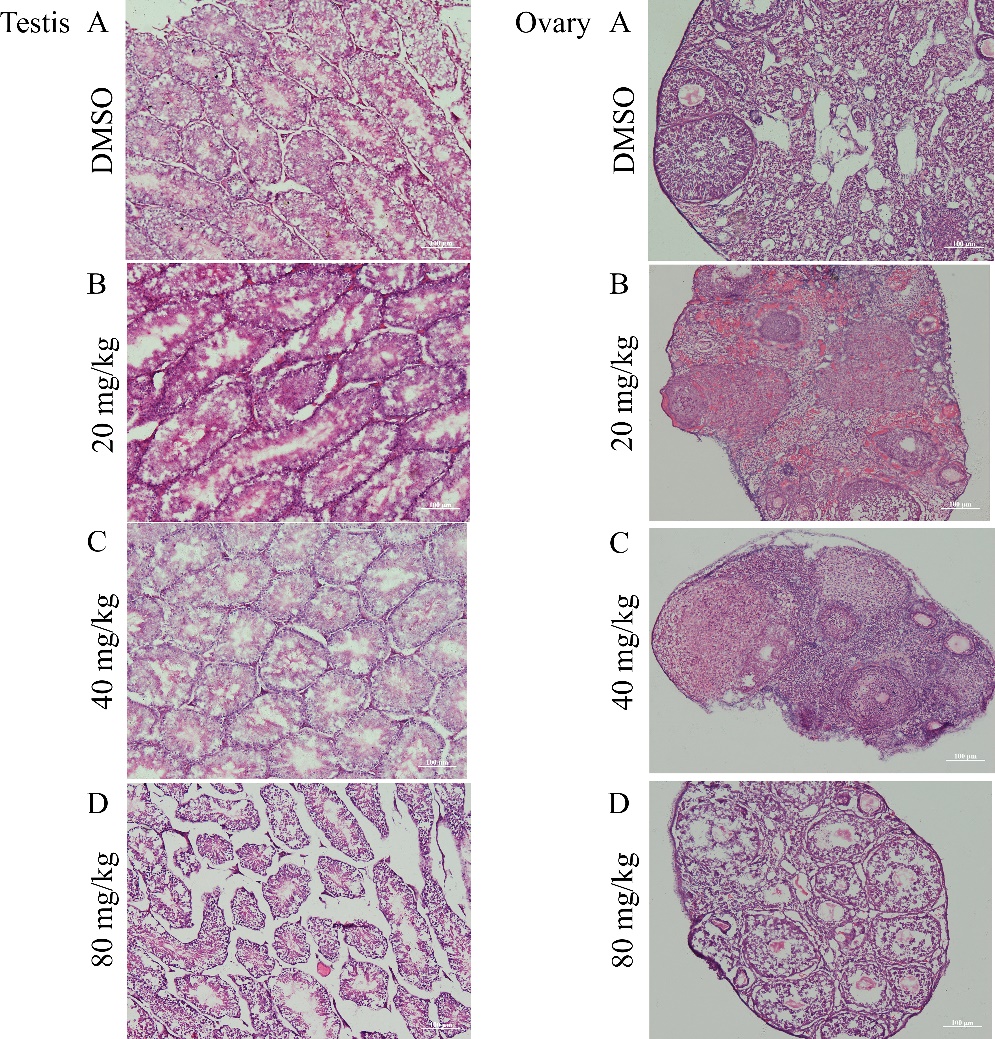


**Figure S1.** Photomicrograph of the testes and ovaries of mice treated with DMSO or gossypol solution of cottonseed powder. A, group treated with DMSO (control group); B, group treated with 20 mg/kg gossypol; C, group treated with 40 mg/kg gossypol; D, group treated with 80 mg/kg gossypol. N.B., Right-side images represent male mice, and the left-side images represent female mice.


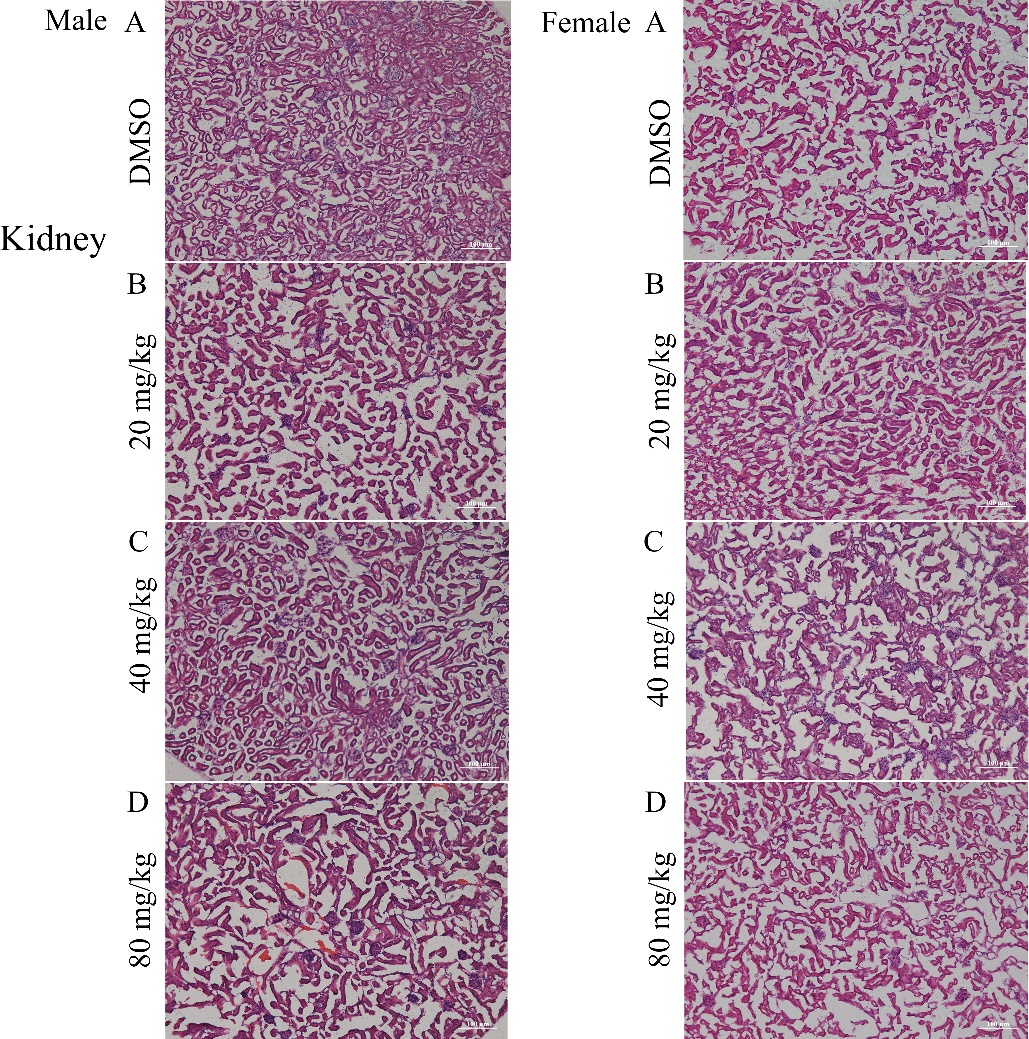


**Figure S2.** Photomicrograph of the kidneys of mice treated with DMSO or gossypol solution of cottonseed powder. A, group treated with DMSO (control group); B, group treated with 20 mg/kg gossypol; C, group treated with 40 mg/kg gossypol; D, group treated with 80 mg/kg gossypol. N.B., Right-side images represent male mice, and the left-side images represent female mice.


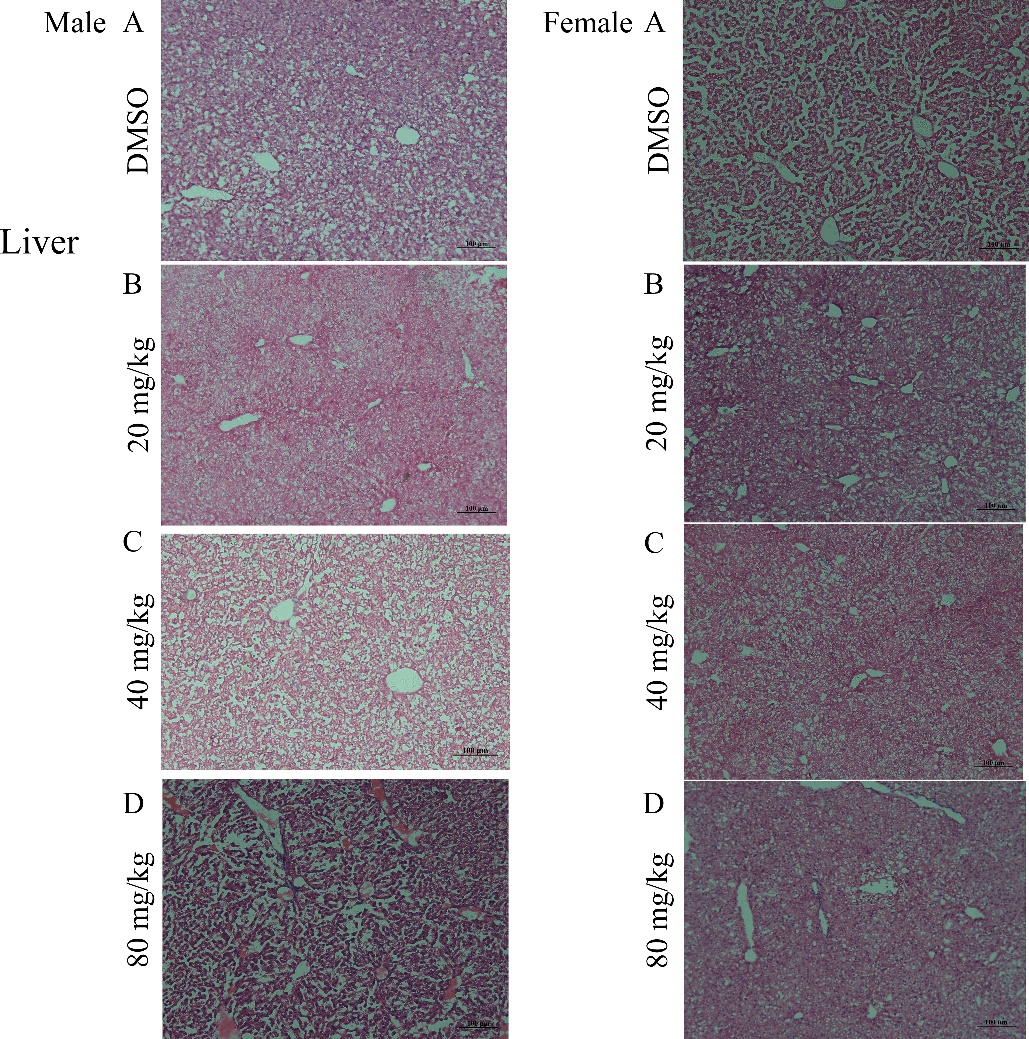


**Figure S3.** Photomicrograph of the liver of a mouse treated with DMSO or gossypol solution of cottonseed powder. A, group treated with DMSO (control group); B, group treated with 20 mg/kg gossypol; C, group treated with 40 mg/kg gossypol; D, group treated with 80 mg/kg gossypol. N.B., Right-side images represent male mice, and the left-side images represent female mice.


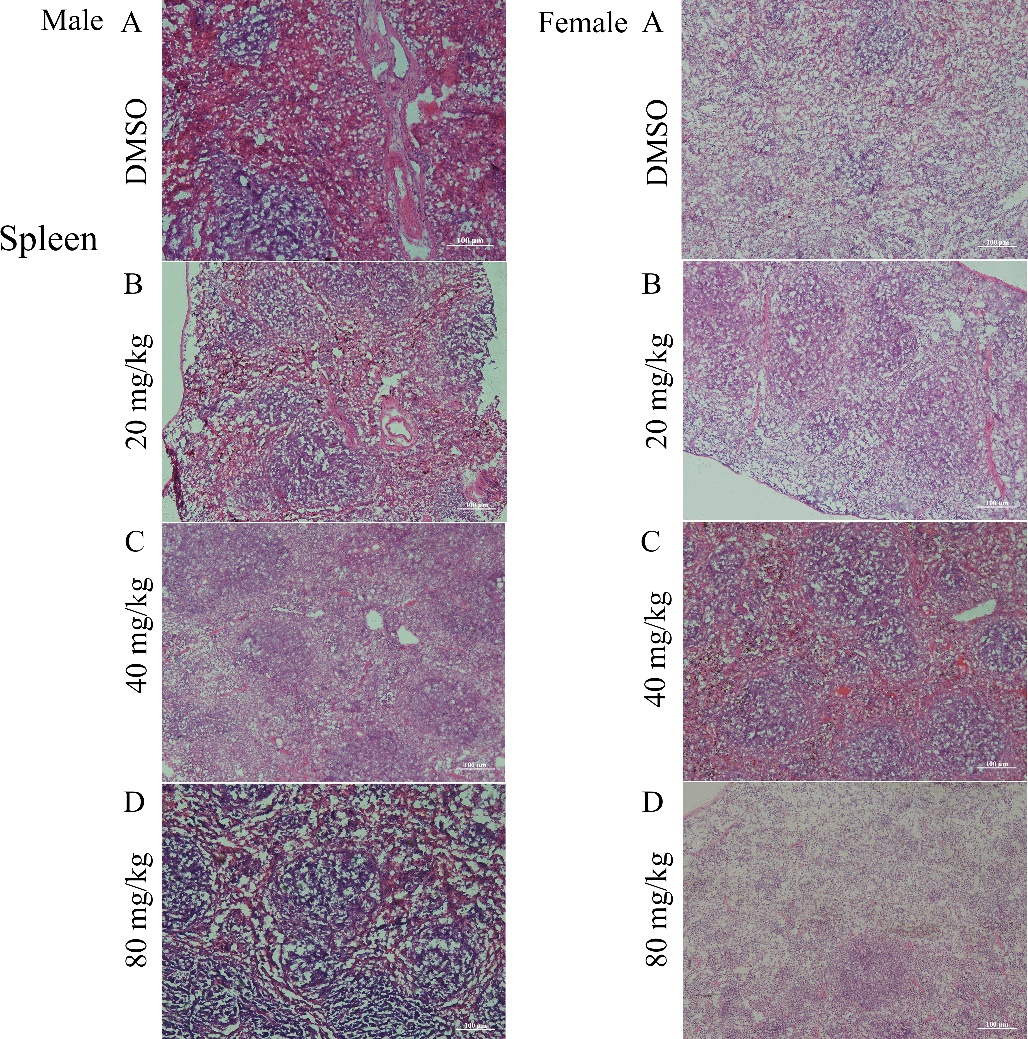


**Figure S4.** Photomicrograph of the spleen of a mouse treated with DMSO or gossypol solution of cottonseed powder. A, group treated with DMSO (control group); B, group treated with 20 mg/kg gossypol; C, group treated with 40 mg/kg gossypol; D, group treated with 80 mg/kg gossypol. N.B., Right-side images represent male mice, and the left-side images represent female mice.


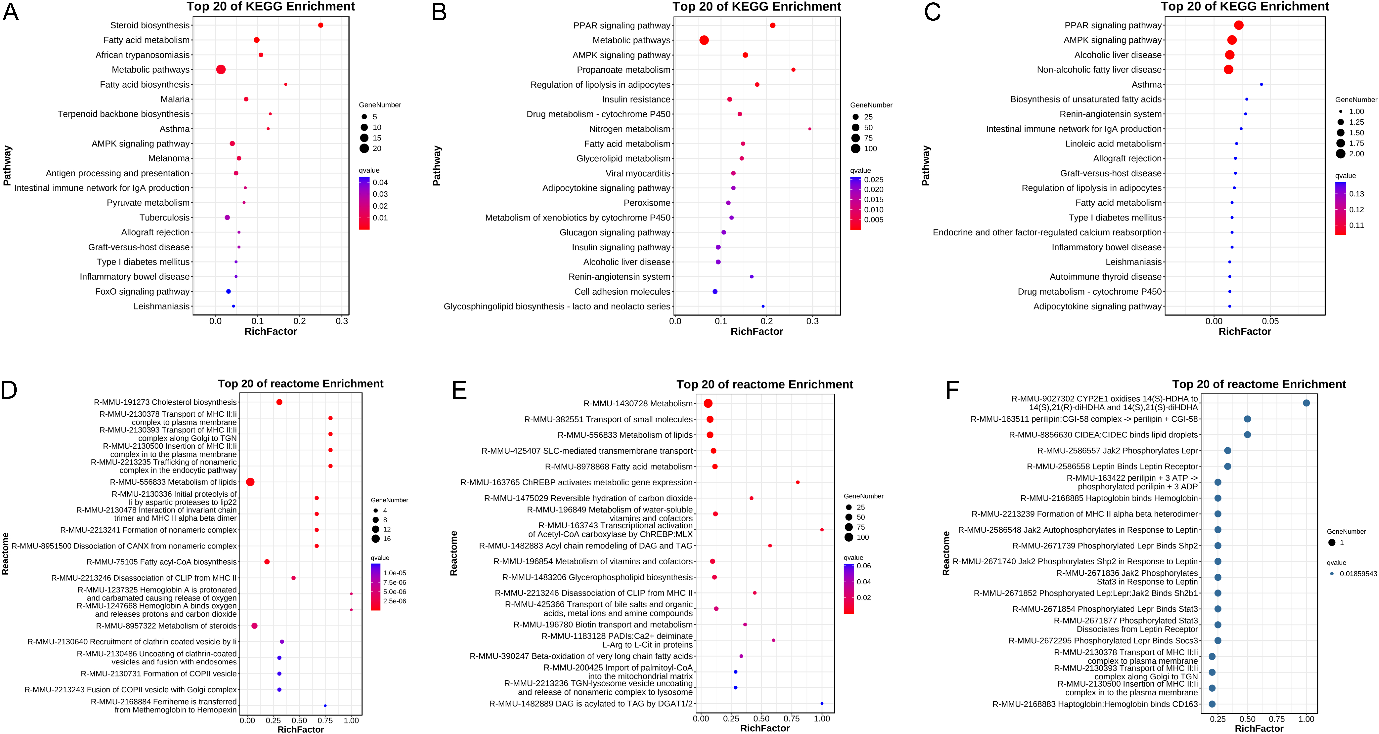


**Figure S5.** KEGG pathway enrichment analysis and reactome enrichment analysis. A, Pathway enrichment in the ovary; B, pathway enrichment in the liver; C, pathway enrichment in the testis; D, reactome enrichment in the ovary; B, reactome enrichment in the liver; C, reactome enrichment in the testis.
