# Supplementary material for: Histopathological examination and transcriptomic profiling reveal gossypol toxicity-responsive genes related to fertility in mice
Source: Front Pharmacol. 2025 Aug 29;16:1654299. doi: 10.3389/fphar.2025.1654299 (PMC12425930; doi:10.3389/fphar.2025.1654299)
Supplement: Supplementary file 2 [file Supplementaryfile2.docx]

**Supplementary Tables and Captions**

**Table S1:** List of primers for RT-qPCR

| **Gene ID** | **Forward primer** | **Reverse primer** |
| --- | --- | --- |
| *ENSMUSG00000015843* | ACTGTCGCTACCAGAAGTGC | TAGAATCCTCTCCACGGGCA |
| *ENSMUSG00000051314* | GCACTGGACCAGAGGAGAAC | TAGGCCGTGAGGATCAAGGA |
| *ENSMUSG00000025488* | AAAAAGCCCCAGCTGGCTAC | TTGGCATGATTCTCGGGGTC |
| *ENSMUSG00000026621* | ACTGAAACGGGTGATGGCTT | GGCGGTAGCTCTTCAGTGTT |
| *ENSMUSG00000040283* | GGGAAGCGTGAACCTACACA | GAGCCCGATCCTGCTACTTC |
| *ENSMUSG00000029195* | TGTTCTGCTGCGAGCTGTTA | TTGGGTTTACCGGACTCACG |
| *ENSMUSG00000030935* | TCCACCACCAACACTAAAGCA | GTGAGAACCTCACGAGTCGG |
| *ENSMUSG00000036144* | GGGATTAGTCGGCTCTGCTC | AGCAGAAGACTTCACGGACG |
| *ENSMUSG00000033849* | TCGTGTTCAATCACTGGCGA | TTCCTTTGCTGCATTGGCAC |
| *ENSMUSG00000066319* | GCCACAGATCTTTCTGGACCC | GTGCCAGGGTTTCACCTCTT |
| *ENSMUSG00000021922* | CCACATCTGGTTGTGACGGA | TCACTTCGAGCCACGAGAAC |
| *ENSMUSG00000045569* | CACCACAATCCTCTACCCTCA | GCTGCTCATCCAGGAATGTTG |
| *ENSMUSG00000048138* | GGGGCTTTCTGGGAAACAGA | TGGGCGATAACCTTCTAAAATGC |
| *ENSMUSG00000032942* | GACCCACGGCCTTCTACAAA | TCAAAACGGAGATTCCCGCA |
| *ENSMUSG00000021708* | CGACCTTCCTGTTCTCCCTG | CAGAGCTAGGAGCCACCCTA |
| *ENSMUSG00000010651* | CTCCGACGTGGTGGTGG | CTTCACGTCCTGGAGAACCG |
| *ENSMUSG00000047822* | GCAGAGCCACCTCTTATGGG | GGTGGCATCCAGGTAGTCTC |
| *ENSMUSG00000029843* | AGCCTACAGCTGCTCTCTCT | GAGGTTTCCTCTCAGGGCAC |
| *ENSMUSG00000018566* | GCAGATCGGCTCTGACGATG | CAATCACCTTCTGTGGGGCA |
| *ENSMUSG00000031972* | CAGAGTCAGAGCAGCAGAAACTA | GGCAAAGCCAGCTTTCACCA |
| *ENSMUSG00000070570* | GTAGTGGCTGCCCAAAGCTA | ATGGCCACTGAGAAACCGAG |
| *ENSMUSG00000027871* | GGCCTGTGTTCAAGCAAGTG | TCTGTTCCTCGTGGCCATTC |
| *ENSMUSG00000025582* | AAAGGGCCAAGATCGAGAGC | AGCTGAAACTTGTCTCCGGG |
| *ENSMUSG00000024617* | AGCCCTAGTTCCCAGCCTAA | GCTCCCTTTCCCAGTTCCTC |
| *ENSMUSG00000027273* | TCCTCCGGAGAAGACAAGGT | ATCTCCTCCAGCTCATTGCG |
| *ENSMUSG00000021670* | GGCTTGGCCTCCATTGAGAT | AGGCCATGCATCCGGAAAA |
| *ENSMUSG00000030302* | CCTGTGAGACCATGGGCAAT | AGCGTCTTGGCATTGATGGA |
| *ENSMUSG00000030827* | GCCATTCACTTTGCCTGAGC | ATCCATTCCATCAGGGCTGC |
| *ENSMUSG00000020932* | ACCTCCAGATCCGAGAAACC | AATGACCTCACCATCCCGCA |
| *ENSMUSG00000018774* | TGTTCAGCTCCAAGCCCAAA | GTACCGTCACAACCTCCCTG |
| Gap | CACACAGGATGGCTTGAAGA | AGGGCAGAATCATCACGAAG |

**Table S2:** Body weight data from mice subjected to oval gavage gossypol treatment for 14 days

| **Male Group** | **DMSO** | **20 mg/kg** | **40 mg/kg** | **80 mg/kg** |
| --- | --- | --- | --- | --- |
| Initial weight | 20.33 + 0.57 | 20.67 + 0.57 | 20.33 + 0.57 | 20.33 + 0.57 |
| 7^th^ day | 22.00 + 1.00 | 20.67 + 2.08 | 20.00 + 1.00 | 20.00 + 1.00 |
| 14^th^ day | 23.00 + 1.73 | 22.00 + 1.00 | 21.33 + 2.08 | 19.67 + 2.08 |
| **Female Group** |  |  |  |  |
| Initial weight | 17.33 + 0.57 | 17.67 + 0.57 | 17.33 + 0.57 | 17.33 + 0.57 |
| 7^th^ day | 17.33 + 0.57 | 17.00 + 2.00 | 18.67 + 0.57 | 16.33 + 0.57 |
| 14^th^ day | 19.00 + 1.00 ^a^ | 19.00 + 1.00 ^a^ | 18.67 + 0.57 ^a^ | 15.67 + 0.57 ^b^ |

**Note:** Values are expressed as the means ± SDs (n=5). Tukey's Studentized Range (HSD) test was used to compare treatment means. means that no letters in common are significantly different at the 5% probability level. If no letters are given, the overall p value was not statistically significant at the 5% probability level.

**Table S3:** Hematology of male mice given cotton gossypol powder solution for 14 days.

| **Parameters** | **DMSO** | **20 mg/kg** | **40 mg/kg** | **80 mg/kg** |
| --- | --- | --- | --- | --- |
| Neutrophil, % | 13.63 + 3.63 ^b^ | 21.53 + 1.96 ^a^ | 21.40 + 3.70 ^a^ | 28.83 + 3.88 ^a^ |
| Monocyte, % | 3.57 + 0.61 ^b^ | 1.76 + 1.56 ^b^ | 0.57 + 1.52 ^c^ | 9.100 + 1.47 ^a^ |
| Lymphocyte, % | 81.00 + 5.20 ^a^ | 87.67 + 6.93 ^a^ | 83.67 + 2.41 ^a^ | 53.27 + 6.77 ^b^ |
| RBC, × 10^12^/L | 9.63 + 0.50 | 9.45 + 0.47 | 9.45 + 0.23 | 9.14 + 0.46 |
| MCV, fL | 50.63 + 1.82 | 50.33 + 1.20 | 51.97 + 0.56 | 51.33 + 0.47 |
| PCT, % | 48.10 + 2.65 | 53.13 + 2.65 | 47.47 + 0.61 | 46.93 + 2.01 |
| PLT, × 10^9^/L | 566.00 + 104.12 | 814.67 + 188.78 | 656.67 + 108.84 | 585.67 + 125.03 |
| Eosinophil, % | 0.00 + 0.00 | 0.033 + 0.05 | 0.00 + 0.00 | 0.00 + 0.00 |
| Basophil, % | 0.33 + 0.30 | 0.16 + 0.28 | 0.83 + 0.97 | 0.53 + 0.47 |
| Neutrophil, × 10^9^/L | 0.43 + 0.49 | 0.16 + 0.15 | 0.30 + 0.00 | 0.56 + 0.30 |
| Lymphocyte, × 10^9^/L | 1.50 + 0.17 | 1.23 + 0.40 | 1.73 + 0.92 | 0.70 + 0.26 |
| Monocyte, × 10^9^/L | 0.20 + 0.17 | 0.06 + 0.11 | 0.03 + 0.05 | 0.16 + 0.11 |
| WBC, × 10^9^/L | 2.13 + 0.49 | 1.53 + 0.40 | 2.03 + 0.92 | 1.43 + 0.20 |
| HGB, g/L | 146.33 + 10.78 | 160.33 + 2.51 | 150.00 + 6.92 | 143.33 + 5.50 |
| MCH, pg | 15.20 + 0.62 | 15.76 + 0.30 | 15.83 +0.05 | 15.70 + 0.20 |
| HCT, % | 48.10 + 2.65 | 51.73 + 4.67 | 47.46 + 0.61 | 46.93 + 2.01 |
| MCHC, g/L | 304.33 + 12.67 | 297.00 + 6.24 | 304.00 + 3.00 | 305.00 + 4.58 |
| RDW-SD, fL | 7.03 + 1.09 | 6.46 + 1.99 | 6.96 + 0.30 | 6.80 + 1.10 |
| RDW-CV, % | 17.60 + 0.79 | 16.63 + 1.91 | 17.53 + 0.41 | 18.46 + 0.66 |
| PDW, fL | 7.26 + 0.15 | 9.60 + 4.34 | 7.00 + 0.30 | 6.90 + 0.36 |
| MPV, fL | 7.03 + 0.15 | 6.46 + 0.77 | 6.96 + .015 | 6.80 + 0.10 |
| P-LCR, % | 3.60 + 0.20 | 4.00 + 0.91 | 4.56 + 0.77 | 3.70 + 0.75 |
| Eosinophil, × 10^9^/L | 0.00 + 0.00 | 0.00 + 0.00 | 0.00 + 0.00 | 0.00 + 0.00 |
| Basophil, × 10^9^/L | 0.00 + 0.00 | 0.00 + 0.00 | 0.00 + 0.00 | 0.00 + 0.00 |

**Note:** Values are expressed as the means ± SDs (n=5). Tukey's Studentized Range (HSD) test was used to compare treatment means. means that no letters in common are significantly different at the 5% probability level. If no letters are given, the overall p value was not statistically significant at the 5% probability level. Abbreviations: fL, femtoliters; HCT, hematocrit; HGB, hemoglobin; MCH, mean corpuscular hemoglobin; MCHC, mean corpuscular hemoglobin concentration; MCV, mean corpuscular volume; MPV, mean plasma volume; PCT, procalcitonin test; RBC, red blood cell; WBC, white blood cell; PDW, platelet distribution width; RDW-SD, red cell distribution width; RDW-CV, red cell distribution width–corpuscular volume; P-LCR, platelet–large cell ratio.

**Table S4:** Hematology of female mice given cotton gossypol powder solution for 14 days

| **Parameters** | **DMSO** | **20 mg/kg** | **40 mg/kg** | **80 mg/kg** |
| --- | --- | --- | --- | --- |
| Neutrophil, % | 8.16 + 1.68 ^b^ | 14.70 + 2.94 ^a^ | 14.70 + 5.20 ^a^ | 37.10 + 16.99 ^a^ |
| Monocyte, % | 1.66 + 0.41 ^b^ | 0.30 + 0.100 ^b^ | 2.56 + 1.00 ^b^ | 10.67 + 4.52 ^a^ |
| Lymphocyte, % | 84.87 + 8.03 ^a^ | 94.00 + 5.46 ^a^ | 71.87 + 8.13 ^b^ | 62.30 + 20.95 ^b^ |
| RBC, x 10^12^/L | 9.80 + 0.34 ^a^ | 9.47 + 0.11 ^a^ | 10.87 + 0.92 ^a^ | 8.76 + 0.40 ^b^ |
| MCV, fL | 49.97 + 0.55 | 51.50 + 0.87 | 50.93 + 0.80 | 51.86 + 1.95 |
| PCT, % | 48.43 + 1.34 | 51.33 + 1.00 | 53.93 + 3.35 | 45.63 + 3.89 |
| PLT, x 10^9^/L | 538.33 + 260.04 | 753.67 + 70.60 | 736.00 + 30.51 | 674.67 + 58.75 |
| Eosinophil, % | 0.00 + 0.00 | 0.06 + 0.11 | 0.00 + 0.00 | 0.10 + 0.17 |
| Basophil, % | 0.16 + 0.28 | 0.36 + 0.35 | 0.80 + 0.36 | 0.10 + 0.17 |
| Neutrophil, x 10^9^/L | 0.16 + 0.05 ^b^ | 0.10 + 0.17 ^b^ | 0.23 + 0.11 ^a^ | 0.46 + 0.05 ^a^ |
| Lymphocyte, x 10^9^/L | 1.76 + 0.68 | 1.60 + 0.45 | 0.80 + 0.26 | 1.20 + 1.12 |
| Monocyte, x 10^9^/L | 0.03 + 0.05 | 0.20 + 0.00 | 0.06 + 0.05 | 0.10 + 0.10 |
| WBC, x 10^9^/L | 2.00 + 0.72 | 2.20 + 0.34 | 2.27 + 1.76 | 1.97 + 0.96 |
| HGB, g/L | 156.00 + 6.08 | 149.33 + 2.30 | 176.67 + 8.32 | 137.67 + 10.26 |
| MCH, pg | 15.93 + 0.05 | 15.23 + 0.60 | 15.83 + 0.47 | 15.63 + 0.41 |
| HCT, % | 48.43 + 1.34 | 51.33 + 1.00 | 55.66 + 4.86 | 45.63 + 3.89 |
| MCHC, g/L | 322.33 + 4.50 ^a^ | 304.00 + 2.64 ^b^ | 309.33 + 2.88 ^b^ | 304.67 + 1.15 ^b^ |
| RDW-SD, fL | 24.13 + 0.80 ^b^ | 29.20 + 2.28 ^a^ | 26.30 + 1.93 ^a^ | 26.13 + 1.52 ^a^ |
| RDW-CV, % | 17.56 + 0.40 | 17.76 + 2.85 | 19.53 + 0.75 | 17.76 + 0.72 |
| PDW, fL | 9.13 + 0.46 | 7.27 + 1.88 | 8.46 + 0.40 | 7.60 + 0.69 |
| MPV, fL | 7.67 + 0.05 | 9.70 + 3.98 | 7.47 + 0.05 | 7.16 + 0.23 |
| P-LCR, % | 8.10 + 1.15 ^a^ | 5.73 + 0.47 ^b^ | 6.20 + 0.60 b | 5.57 + 0.75 ^b^ |
| Eosinophil, x 10^9^/L | 0.00 + 0.00 | 0.01 + 0.01 | 0.00 + 0.00 | 0.00 + 0.00 |
| Basophil, x 10^9^/L | 0.02 + 0.04 | 0.00 + 0.00 | 0.01 + 0.00 | 0.00 + 0.00 |

**Note:** Values are expressed as the means ± SDs (n=5). Tukey's Studentized Range (HSD) test was used to compare treatment means. means that no letters in common are significantly different at the 5% probability level. If no letters are given, the overall p value was not statistically significant at the 5% probability level. Abbreviations: fL, femtoliters; HCT, hematocrit; HGB, hemoglobin; MCH, mean corpuscular hemoglobin; MCHC, mean corpuscular hemoglobin concentration; MCV, mean corpuscular volume; MPV, mean plasma volume; PCT, procalcitonin test; RBC, red blood cell; WBC, white blood cell; PDW, platelet distribution width; RDW-SD, red cell distribution width; RDW-CV, red cell distribution width–corpuscular volume; P-LCR, platelet–large cell ratio.
